# Multigenomic Delineation of *Plasmodium* Species of the *Laverania* Subgenus Infecting Wild-Living Chimpanzees and Gorillas

**DOI:** 10.1093/gbe/evw128

**Published:** 2016-06-11

**Authors:** Weimin Liu, Sesh A. Sundararaman, Dorothy E. Loy, Gerald H. Learn, Yingying Li, Lindsey J. Plenderleith, Jean-Bosco N. Ndjango, Sheri Speede, Rebeca Atencia, Debby Cox, George M. Shaw, Ahidjo Ayouba, Martine Peeters, Julian C. Rayner, Beatrice H. Hahn, Paul M. Sharp

**Affiliations:** ^1^Department of Medicine, Perelman School of Medicine, University of Pennsylvania; ^2^Department of Microbiology, Perelman School of Medicine, University of Pennsylvania; ^3^Institute of Evolutionary Biology, and Centre for Immunity, Infection and Evolution, University of Edinburgh, United Kingdom; ^4^Faculty of Sciences, University of Kisangani, Democratic Republic of the Congo; ^5^Sanaga-Yong Chimpanzee Rescue Center, IDA-Africa, Portland, Oregon; ^6^Tchimpounga Chimpanzee Rehabilitation Center, Pointe-Noire, Republic of the Congo; ^7^Africa Programmes, Jane Goodall Institute, Vienna, Virginia; ^8^UMI 233, Institut de Recherche pour le Développement (IRD), INSERM U1175, and University of Montpellier, France; ^9^Malaria Programme, Wellcome Trust Sanger Institute, Wellcome Genome Campus, Hinxton, Cambridge, UK

**Keywords:** *Laverania*, *Plasmodium* parasites infecting chimpanzees and gorillas, cryptic *Plasmodium* species, single genome sequencing, *P. falciparum*

## Abstract

*Plasmodium falciparum*, the major cause of malaria morbidity and mortality worldwide, is only distantly related to other human malaria parasites and has thus been placed in a separate subgenus, termed *Laverania*. Parasites morphologically similar to *P. falciparum* have been identified in African apes, but only one other *Laverania* species, *Plasmodium reichenowi* from chimpanzees, has been formally described. Although recent studies have pointed to the existence of additional *Laverania* species, their precise number and host associations remain uncertain, primarily because of limited sampling and a paucity of parasite sequences other than from mitochondrial DNA. To address this, we used limiting dilution polymerase chain reaction to amplify additional parasite sequences from a large number of chimpanzee and gorilla blood and fecal samples collected at two sanctuaries and 30 field sites across equatorial Africa. Phylogenetic analyses of more than 2,000 new sequences derived from the mitochondrial, nuclear, and apicoplast genomes revealed six divergent and well-supported clades within the *Laverania* parasite group. Although two of these clades exhibited deep subdivisions in phylogenies estimated from organelle gene sequences, these sublineages were geographically defined and not present in trees from four unlinked nuclear loci. This greatly expanded sequence data set thus confirms six, and not seven or more, ape *Laverania* species, of which *P. reichenowi*, *Plasmodium gaboni*, and *Plasmodium billcollinsi* only infect chimpanzees, whereas *Plasmodium praefalciparum, Plasmodium adleri*, and *Pladmodium blacklocki* only infect gorillas. The new sequence data also confirm the *P. praefalciparum* origin of human *P. falciparum*.

## Introduction

Of the five *Plasmodium* species known to commonly infect humans, *Plasmodium falciparum* is by far the most pathogenic, causing over 200 million clinical cases of malaria and over half a million malaria-related deaths annually (White et al. 2014). Given this public health impact, there is an urgent need to elucidate new strategies to combat this pathogen. One avenue for new discovery is to study *P. falciparum* in the context of its closest simian parasite relatives. For nearly a century it has been known that chimpanzees (*Pan troglodytes*) and western gorillas (*Gorilla gorilla*) harbor parasites that are morphologically indistinguishable from *P. falciparum* (Reichenow 1920; Blacklock and Adler 1922; Adler 1923), but only one such species, *Plasmodium reichenowi*, has been formally described (Coatney et al. 1971). Within the last decade, studies of both captive and wild-living African apes have produced sequences that imply a multitude of genetically diverse parasites, pointing to the existence of additional ape *Plasmodium* species (Ollomo et al. 2009; Rich et al. 2009; Duval et al. 2010; Krief et al. 2010; Liu et al. 2010a; Prugnolle et al. 2010). Initially one new species, *Plasmodium gaboni*, was proposed on the basis of divergent mitochondrial (mt) DNA sequences amplified from the blood of two wild-born chimpanzees (Ollomo et al. 2009). A further two species, termed *Plasmodium billcollinsi* and *Plasmodium billbrayi*, were suggested based on mtDNA and nuclear gene sequences, also obtained from chimpanzee samples (Krief et al. 2010). Finally, noninvasive testing of wild-living gorillas revealed still more parasite lineages, one of which was genetically nearly identical to human *P. falciparum* (Liu et al. 2010a; Prugnolle et al. 2010). Collectively, these studies indicated a much larger variety of ape parasites than previously appreciated (Liu et al. 2010a; Rayner et al. 2011).

Early studies showed that *P. falciparum* and *P. reichenowi* are quite distinct from all other *Plasmodium* species, which prompted their classification into a separate subgenus, termed *Laverania* (Bray 1963). However, microscopic studies of *P. falciparum* and *P. reichenowi* (Coatney et al. 1971), and more recently *P. gaboni* (Ollomo et al. 2009), failed to identify morphological differences. The endangered species status of chimpanzees and gorillas precludes the types of life history studies traditionally used for the taxonomic description of *Plasmodium* species, and so these cryptic *Laverania* species will likely have to be classified on the basis of genetic data (Perkins 2000). Amplifying parasite DNA sequences from fecal samples from a large number of wild-living chimpanzees and gorillas, we previously identified six well-supported chimpanzee- (C1–C3) and gorilla (G1–G3)-specific clades. Since each of these clades was at least as divergent from the others as *P. falciparum* was from *P. reichenowi*, we proposed six ape *Laverania* species, comprising the chimpanzee parasites *P. reichenowi* (C1), *P. gaboni* (C2), and *P. billcollinsi* (C3), and the gorilla parasites *Plasmodium praefalciparum* (G1), *P. adleri* (G2), and *Plasmodium blacklocki* (G3) (Liu et al. 2010a; Rayner et al. 2011). Sequences from the fourth proposed chimpanzee parasite species, *P. billbrayi* (Krief et al. 2010), were very closely related to sequences from *P. gaboni* parasites, such that we did not consider these to represent two separate species (Liu et al. 2010a; Rayner et al. 2011). Nevertheless, others continue to recognize both (Prugnolle et al. 2011; Snounou et al. 2011; Pacheco et al. 2013; Boundenga et al. 2015; Herbert et al. 2015; Makanga et al. 2016), implying a total of seven *Laverania* species infecting apes, and some have split these lineages even further, suggesting at least ten new *Laverania* species (Zilversmit and Awadalla 2011; Zilversmit et al. 2013).

Perspectives on the number of *Laverania* species could have been influenced by limited geographic sampling and by the fact that most of the existing genetic information derives from the mitochondrial genome, a small, uniparentally inherited, nonrecombining molecule that may not be ideal for delineating species (Galtier et al. 2009). In an attempt to resolve these issues, we have amplified parasite sequences from a large number of additional chimpanzee and gorilla samples collected in geographic regions that have previously been underrepresented. Testing nearly 250 new *Laverania* positive specimens, we almost tripled the number of existing *Laverania* sequences. Importantly, we have analyzed multiple unlinked loci from the parasite nuclear genome. Our results indicate that there are six, and not seven (or more), clearly defined ape *Laverania* species, and that mitochondrial sequences alone are not sufficient to delineate cryptic *Plasmodium* species reliably.

## Expanding the *Laverania* Sequence Database

**Fig. 1.**
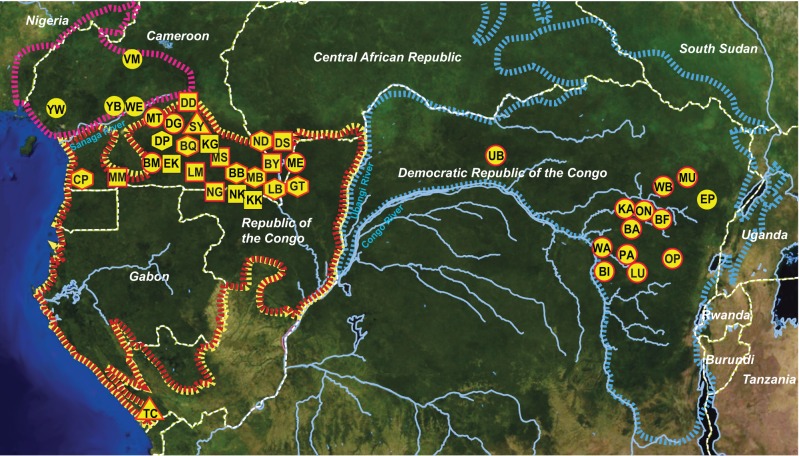
Locations of sites where *Laverania*-positive apes were sampled. The ranges of ape species and subspecies are indicated: Nigeria-Cameroonian chimpanzee (*Pan t. ellioti*) (magenta), central chimpanzees (*Pan t. troglodytes*) (red), eastern chimpanzees (*Pan t. schweinfurthii*) (blue), and western gorillas (*G. g. gorilla*) (yellow). Circles, squares, and octagons identify field sites where fecal samples were collected from chimpanzees, gorillas or both, respectively (supplementary table S1, Supplementary Material online); triangles indicate two chimpanzee sanctuaries. A red border indicates all field sites from which new *Laverania* sequences were generated for this study (supplementary table S2, Supplementary Material online). Country borders (white) and major rivers (blue) are indicated.

To increase the geographic representation of *Laverania* sequences, we selected 248 samples from more than 100 sanctuary and wild-living apes initially surveyed to characterize the molecular epidemiology of primate lentiviruses (Keele et al. 2006; Neel et al. 2010; Li et al. 2012; D'arc et al. 2015). Samples from eastern chimpanzees (*Pan t. schweinfurthii*) and western lowland gorillas (*G. g. gorilla*) were specifically targeted, because these were underrepresented in previous studies of *Laverania* infections. All specimens were *Laverania* positive as determined by either diagnostic or limiting dilution polymerase chain reaction (PCR) amplification of mtDNA sequences. Fecal samples (*n* = 216) were derived from nonhabituated ape populations at 30 field sites across central Africa, including six locations where chimpanzees and gorillas have not previously been screened for *Plasmodium* infections (fig. 1), while blood samples (*n* = 32) were obtained from 25 chimpanzees at two sanctuaries (Sanaga Yong Wildlife Rescue Center, SY, and Tchimpounga Chimpanzee Rehabilitation Center, TC), and from one gorilla bushmeat sample (SA) of unknown geographic origin (supplementary tables S1 and S2, Supplementary Material online). Although the sanctuary chimpanzees were almost all healthy at the time of sampling, their blood samples had higher parasite loads than fecal samples and usually contained one predominant *Laverania* species. In contrast, fecal samples, which contain DNA from both liver and blood stage parasites, contained multiple *Laverania* species (Liu et al. 2010a; Abkallo et al. 2014).

Having assembled a geographically diverse sample set, we next aimed to amplify regions of the mitochondrial, apicoplast, and nuclear parasite genomes. Apes can be simultaneously infected with multiple *Plasmodium* species, and so conventional (bulk) PCR is not appropriate to generate *Plasmodium* sequences for phylogenetic analyses, because *Taq* polymerase has the propensity to switch templates and to generate in vitro recombinants (Liu et al. 2010a). In contrast, limiting dilution PCR, also termed single-genome amplification (SGA), precludes such *Taq*-induced artifacts and provides a proportional representation of the *Laverania* variants present in the sample (Liu et al. 2010b). Using this approach, we amplified a 956-bp fragment spanning most of the mitochondrial cytochrome B (*cytB*) gene. From 451 new *cytB* sequences, exclusion of identical sequences from the same sample yielded 259 new *cytB* haplotypes, bringing the total number to 709 (table 1).

**Table 1:** Single-Genome Amplification Derived Ape *Laverania* Sequences

Species/Subspecies	Fecal Samples	Mitochondrial		Nuclear								Apicoplast	
		*cytB*^a^		*eba165*^a^		*eba175*^a^		*p47*^a^		*ldh*^a^		*clpM*^a^	
		New (hap)^b^	Pub (hap)^b^	New (hap)^b^	Pub (hap)^b^	New (hap)^b^	Pub (hap)^b^	New (hap)^b^	Pub (hap)^b^	New (hap)^b^	Pub (hap)^b^	New (hap)^b^	Pub (hap)^b^
Nigeria-Cameroon chimpanzee *(P. t. ellioti)*	22	31 (4)	205 (51)	24 (9)	7 (2)	19 (8)	NA	42 (13)	NA	139 (10)	NA	32 (7)	21 (9)
Central chimpanzee *(Pan t. troglodytes)*	76	38 (17)	483 (157)	87 (19)	43 (18)	62 (19)	29 (16)	184 (42)	NA	283 (27)	18 (15)	172 (32)	53 (31)
Eastern chimpanzee *(Pan t. schweinfurthii)*	118	214 (144)	98 (55)	40 (25)	7 (6)	20 (14)	13 (9)	68 (31)	NA	3 (3)	16 (14)	255 (134)	22 (12)
Western lowland gorilla *(G. g. gorilla)*	126	168 (94)	415 (187)	15 (7)	15 (15)	6 (6)	21 (13)	43 (28)	NA	35 (8)	12 (11)	85 (37)	27 (17)
Total	342	451 (259)	1201 (450)	166 (60)	72 (41)	107 (47)	63 (38)	337 (114)	NA	460 (48)	46 (40)	544 (210)	123 (69)

Note.—NA, not available.

^a^Single-template-amplified regions of *Laverania* mitochondrial (*cytB*), nuclear (*eba165, eba175, p47, and ldh*), and apicoplast (*clpM*) genes (the *clpM* gene, which encodes the Clp chaperone PfC10_API0060, has previously been called *clpC;* Liu et al. 2010a).

^b^Number of newly derived (New) and previously published (Pub) ape *Laverania* sequences (Liu et al. 2010a; Wanaguru et al. 2013; Sundararaman et al. 2016), with bracket indicating distinguishable haplotypes (hap).

**Fig. 2.**
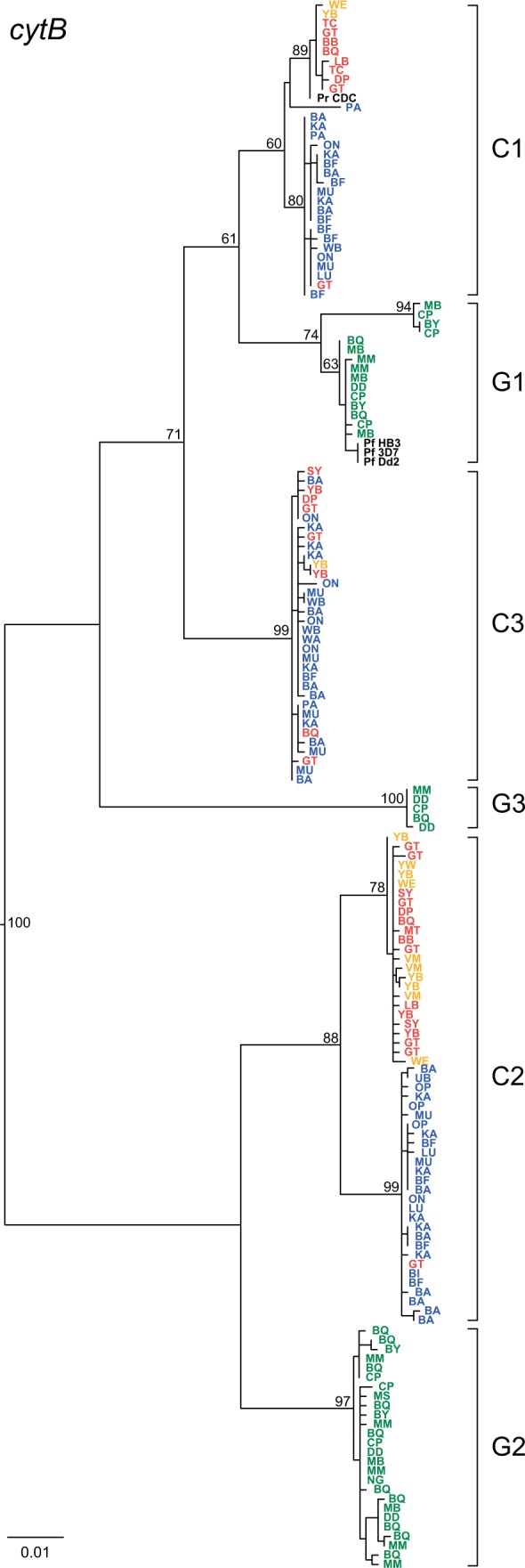
Maximum likelihood phylogeny of *Laverania* mitochondrial DNA sequences. A subset of 165 SGA-derived *cytB* sequences (956 bp) is shown; only distinct haplotypes per field site are shown (the full set of 709 SGA-derived *cytB* sequences is shown in supplementary fig. S1, Supplementary Material online). Sequences are color coded to indicate host: chimpanzees (*Pan t. ellioti* in orange, *Pan t. troglodytes* in red, and *Pan t. schweinfurthi* in blue), or gorillas (*G. g. gorilla* in green), with a two-letter code for the field site of origin (fig. 1). Reference sequences for human *P. falciparum* and chimpanzee *P. reichenowi* are in black. C1, C2, and C3 represent the chimpanzee parasite species *P. reichenowi, P. gaboni*, and *P. billcollinsi*; G1, G2, and G3 represent the gorilla parasite species *P. praefalciparum, P. adleri*, and *P. blacklocki*. Bootstrap values are shown for major nodes only (the scale bar represents 0.01 substitutions per site).

Phylogenetic analyses of the newly derived *cytB* sequences showed that they clustered in six divergent and well-supported clades as reported previously (Liu et al. 2010a). Three of these clades (C1–C3) included only samples from chimpanzees, whereas the other three (G1–G3) included only samples from gorillas (fig. 2). Two of the chimpanzee parasite clades (C1 and C2) contained deep subdivisions, correlating with the geographical origin of the samples: in both cases, one sublineage contained only sequences from Nigerian–Cameroonian (*Pan t. ellioti*) and central (*Pan t. troglodytes*) chimpanzees from west central Africa (fig. 2; orange and red, respectively), while the other was almost exclusively comprised of parasite sequences from eastern chimpanzees (*P. t. schweinfurthii*) from the Democratic Republic of the Congo (fig. 2; blue). These subdivisions were apparent in our previous survey (Liu et al. 2010a), but at the time we had only relatively few samples from *P. t. schweinfurthii*, especially within the C2 clade. The many additional *P. t. schweinfurthii cytB* haplotypes obtained here (supplementary fig. S1*a* and *e*, Supplementary Material online) strongly reinforce this phylogeographic clustering. The clades of eastern chimpanzee-derived sequences within C1 and C2 each contain only a single sequence from a central chimpanzee sample, in both cases from the GT field site; GT is located towards the east of the *P. t. troglodytes* range, closest to the range of *P. t. schweinfurthii* (fig. 1). In addition, one new *cytB* sequence from a *P. t. schweinfurthii* sample (PApts368) fell within the C1 clade but did not cluster significantly with either of the two major sublineages (fig. 2). In contrast, sequences within the third chimpanzee parasite clade (C3) did not cluster according to host subspecies or geographic origin (fig. 2). As shown previously (Liu et al. 2010a; Sundararaman et al. 2013), geographically diverse representatives of human *P. falciparum* clustered within the radiation of G1 parasites (fig. 2).

## Phylogenetic Analyses of *Laverania* Nuclear and Apicoplast Gene Sequences

**Fig. 3.**
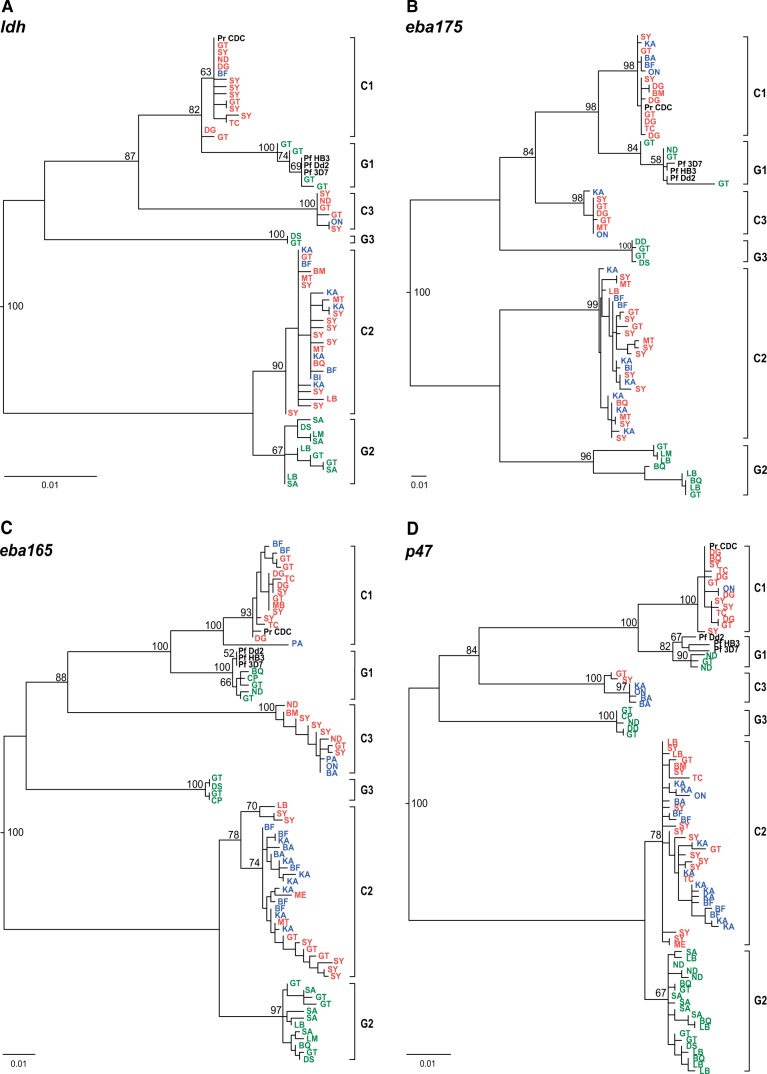
Maximum likelihood phylogenies of *Laverania* nuclear gene sequences. (*A*) *ldh* (772 bp), (*B*) *eba175* (397 bp), (*C*) *eba165* (790 bp), and (*D*) *p47* (800 bp) gene sequences. Only distinct haplotypes per field site are shown (the full sets of sequences are shown in supplementary figs. S2–S5, Supplementary Material online). Sequences and clades are colored and labeled as in figure 2. Numbers on branches indicate percent bootstrap support. The scale bars indicate 0.01 substitutions per site.

To ascertain whether the phylogenies obtained from mtDNA sequences were an accurate reflection of parasite relationships, we amplified fragments from four unlinked nuclear loci (table 1). These included portions of genes encoding lactate dehydrogenase (*ldh;* 772 bp), the erythrocyte binding antigens 165 (*eba165;* 790 bp) and 175 (*eba175;* 397 bp), and the gametocyte surface protein P47 (*p47;* 800 bp). A limited number of *ldh*, *eba165*, and *eba175* sequences have previously been described (Liu et al. 2010a; Wanaguru et al. 2013; Sundararaman et al. 2016), but the *p47* locus, encoding a determinant of mosquito host specificity (Molina-Cruz et al. 2015), has not yet been characterized for ape *Laverania* species (table 1). Overall, more than 1,000 new nuclear gene sequences were generated from either blood or fecal samples (supplementary table S3, Supplementary Material online). Phylogenetic analyses of these sequences again pointed to six major clades within the *Laverania*, corresponding to the three chimpanzee and three gorilla specific clades found in the mtDNA phylogeny (fig. 3). Although the *eba175* sequences were quite short, the overall topologies of all four nuclear gene phylogenies were identical to the *cytB* tree: the C1 and G1 clades clustered together, as did the C2 and G2 clades, with C3 and G3 branching between these two pairs. Although all of these phylogenies were midpoint rooted, the placement of the root of the *Laverania cytB* tree corresponds to that found when more distant *Plasmodium* species are included as outgroups (Liu et al. 2010a). With midpoint rooting, the C2-G2 pair consistently grouped on one side of the root (figs. 2 and 3). The six major clades were each monophyletic in all four nuclear gene trees, with the single exception of the C1 clade in the *ldh* tree (fig. 3A). This lower resolution for *ldh* probably results from the lower level of divergence among the *ldh* sequences (as is apparent from the scale bar). For all four nuclear loci, geographically diverse representatives of human *P. falciparum* clustered with G1 (fig. 3).

The geographically based subdivisions within the C1 and C2 clades, which were apparent in the mtDNA phylogeny, were not seen in the nuclear gene trees (fig. 3). Although the C1 clades contained only few sequences from eastern chimpanzees (blue), there was no evidence that these clustered apart from those from central chimpanzees (red). The C2 clades comprised the largest number of parasite sequences and exhibited considerable substructure; however, again sequences from samples from the different chimpanzee subspecies were interspersed. Interestingly, as in the mtDNA tree, one *P. t. schweinfurthii* sample (PApts368) yielded a C1-related *eba165* sequence that was highly divergent from all other C1 sequences (fig. 3C); however, repeated attempts to amplify other nuclear regions from this sample were unsuccessful.

**Fig. 4.**
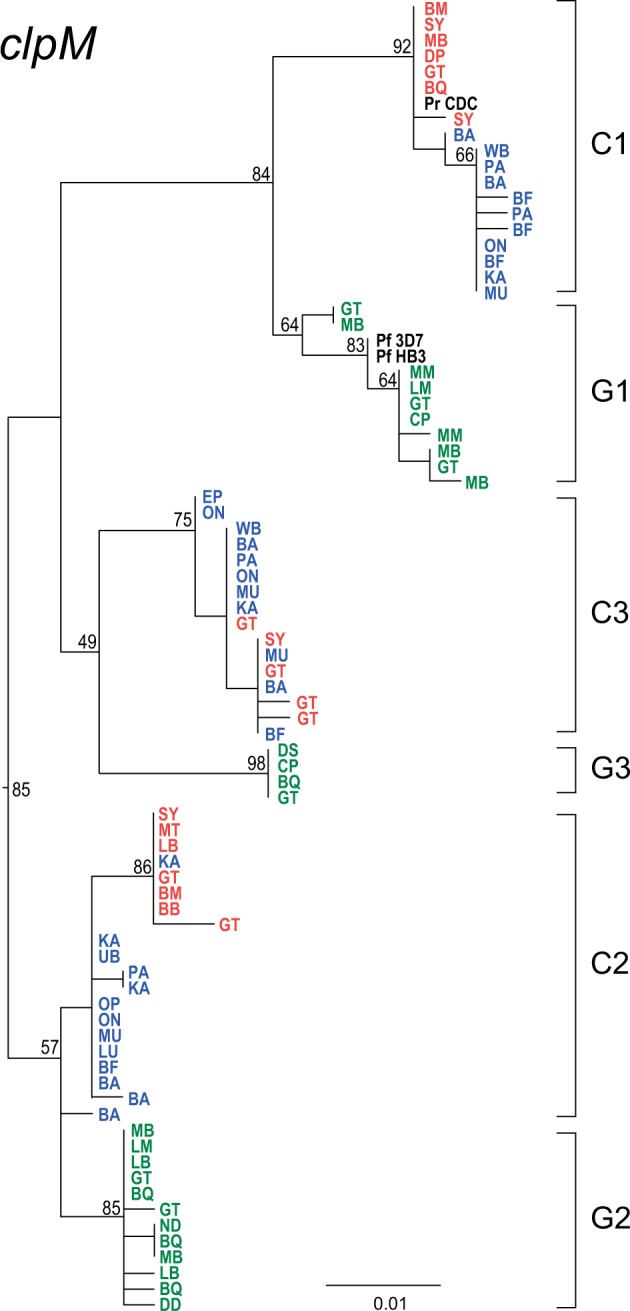
Maximum likelihood phylogeny of *Laverania* apicoplast DNA sequences. A subset of 80 SGA-derived caseinolytic protease M (*clpM*) sequences (390 bp) is shown; only distinct haplotypes per field site are shown (the full set of 227 *clpM* sequences appears in supplementary fig. S6, Supplementary Material online). Sequences and clades are colored and labeled as in figure 2. Numbers on nodes indicate percent bootstrap support. The scale bar represents 0.01 substitutions per site.

Finally, we amplified a 390-bp fragment of the *clpM* gene from the apicoplast genome (note that this gene has previously been referred to as *clpC*; Liu et al. 2010a). Additional SGA-derived *clpM* sequences were obtained from 116 chimpanzee samples (19 from blood and 97 from feces) and 28 gorilla fecal samples. The phylogenetic relationships among these sequences were generally less well supported than for the mtDNA and nuclear loci, which were generally longer and/or faster evolving, and so exhibited more diversity. However, all but one of the sequences fell into the same six major clades (fig. 4). The single exception was a sequence from an eastern chimpanzee sample that fell between the C2 and G2 clades, but the bootstrap support for the branch separating this sequence from C2 was only around 50%. For *clpM*, the relationship among the six clades differed from that found for other genes in two respects: C3 was not more closely related to C1/G1 (but, again, the bootstrap support for this was low), and midpoint rooting would place C1 plus G1 to one side of the root. Because of the relative lack of resolution of the *clpM* tree, it is as yet unclear whether these differences reflect a truly different evolutionary history for the apicoplast genome within *Laverania*. While the short length of the *clpM* fragment, and its relative conservation, resulted in only few nucleotide differences among sequences within the major clades, there was a clear tendency for *clpM* sequences from chimpanzees to cluster based on geographic or host subspecies origin in both C1 and C2, but not C3, clades (fig. 4; supplementary fig. S6, Supplementary Material online). Thus, the apicoplast genome sequences behave similarly to those from the more rapidly evolving mitochondrial genome, while the four nuclear genes do not support any systematic subdivision within the C1 and C2 clades.

## Discussion

Classifying *Laverania* species solely on the basis of genetic information has been controversial (Valkiunas et al. 2011). Although a full taxonomic description would be preferable, it is unrealistic to expect life history and morphological data of the various parasite species to be forthcoming. First, the endangered status of chimpanzees and gorillas precludes invasive studies of any kind, including malaria transmission studies. Second, the high frequency of mixed *Laverania* species infections renders a correlation of parasite morphology with genetic information from the same sample difficult. Although blood samples can be obtained from sanctuary apes for health reasons, these animals are usually subpatently infected and exhibit exceedingly low parasitemia levels (Sundararaman et al. 2016). Thus, laser microdissection of single infected erythrocytes on blood films as proposed (Valkiunas et al. 2011) is not feasible. Given the need to provide a standardized nomenclature of these parasites for future investigations, it seems desirable to arrive at a definition of species based on a comprehensive genetic analysis.

Phylogenetic analyses of more than 3,000 mitochondrial, apicoplast, and nuclear *Laverania* sequences, contributed by this and previous studies, consistently point to the existence of six distinct *Laverania* clades. This, together with the strict host specificity of these clades, even at field sites such as GT where all six *Laverania* clades are cocirculating in sympatric chimpanzee and gorilla populations, indicates that there are strong isolating mechanisms preventing interspecific hybridization. While it has been argued that detection of parasite sequences in either feces or blood is not sufficient to prove productive infection (Valkiunas et al. 2011), the high prevalence of *Laverania* infections in wild ape populations and their widespread geographic distribution across central Africa provide compelling evidence for ongoing transmission of these parasites, even if gametocytes on blood smears have not been formally described. Thus, the overwhelming amount of evidence points to the existence of six distinct *Laverania* species infecting apes.

Although genetic information can inform *Plasmodium* species taxonomy, new species should not be named based on limited sequence data sets. *Plasmodium gaboni* (Ollomo et al. 2009) and *P. billbrayi* (Krief et al. 2010) were each initially proposed based on a small number of mtDNA sequences, from samples of central and eastern chimpanzees, respectively. Subsequent comparisons revealed that these sequences are very closely related, falling within what we term the C2 clade, but the two names have continued to be used by some authors because mtDNA sequences form two distinct sublineages (Prugnolle et al. 2011; Snounou et al. 2011; Pacheco et al. 2013; Boundenga et al. 2015; Herbert et al. 2015; Makanga et al. 2016). By the same criterion, the C1 clade should also be split, because mtDNA again reveals two distinct subclades. However, the additional data reported here shed light on this in several ways. First, within both C1 and C2, it is clear that the mtDNA subdivisions reflect the geographical origins of the samples (fig. 2). Second, sequences from the other organelle genome (the apicoplast) suggest similar phylogeographic splits in both C1 and C2 (fig. 4). Third, in contrast, phylogenies from nuclear DNA sequences fail to show the same subclades within C1 or C2 (fig. 3). (For a quantitative assessment of the difference between organelle and nuclear sequences, with respect to phylogeographic segregation, see supplementary table S4, Supplementary Material online.) These findings are reminiscent of the genetic relationships among the chimpanzee hosts: the central (*P. t. troglodytes*) and eastern (*P. t. schweinfurthii*) chimpanzee subspecies seem phylogenetically distinct based on mtDNA (Gagneux et al. 1999), but less so with respect to nuclear genome variation (Gonder et al. 2011). However, we find no phylogenetic distinction between C1 and C2 parasites infecting the chimpanzees living to the north (*P. t. ellioti*) and south (*P. t. troglodytes*) of the Sanaga River in Cameroon, despite the stronger genetic differentiation between these two host subspecies (Gagneux et al. 1999; Gonder et al. 2011). These two subspecies carry distinct forms of simian foamy viruses (Liu et al. 2008), while simian immunodeficiency viruses infect chimpanzees to the south, but not to the north, of the Sanaga (Sharp and Hahn 2011). Transmission of these viruses requires intimate contact, whereas the mosquito vectors of *Laverania* parasites are evidently able to cross the Sanaga River. Thus, the geographic distance between central and eastern chimpanzees (fig. 1) seems to have led to recent population subdivision in their *Laverania* parasites, which has had a more pronounced effect on organelle genome sequences than on nuclear genes; this is to be expected since these organelle genomes have smaller effective population sizes and will tend to undergo lineage sorting more quickly. However, while mtDNA sequences may reveal incipient subspecies within C1 and C2, nuclear genes suggest that the subclades do not represent distinct species. Thus, there is currently clear evidence for only six *Laverania* species infecting apes, with C1, C2, and C3 clades representing the chimpanzee parasites *P. reichenowi*, *P. gaboni*, and *P. billcollinsi*, and G1, G2, and G3 representing the gorilla parasites *P. praefalciparum, P. adleri*, and *P. blacklocki*, respectively.

The expanded *Laverania* sequence data set reported here also corroborates the origin of human *P. falciparum*. In all analyses, *P. falciparum* clusters closely with gorilla parasites within the G1 clade. Previous studies of mitochondrial gene sequences have shown that *P. falciparum* strains exhibit much less genetic diversity than each of the ape *Laverania* species and that all extant *P. falciparum* strains form a monophyletic lineage within the radiation of the G1 clade of gorilla parasites (Liu et al. 2010a; Sundararaman et al. 2013). These data indicate that parasites within the G1 clade ancestrally infected gorillas and that *P. falciparum* emerged from a more recent gorilla-to-human transmission event (Liu et al. 2010a). The same close relationships between human and gorilla parasites within the G1 clade are seen for nuclear (fig. 3) and apicoplast (fig. 4) gene sequences, although the relationships are generally less well resolved than with mtDNA sequences (fig. 2). Although *P. falciparum* has been detected in a small number of captive chimpanzees and bonobos living in close proximity of humans (Duval et al. 2010; Krief et al. 2010), none of the 216 ape fecal samples characterized in this study (supplementary table S1, Supplementary Material online) contained *P. falciparum* sequences, indicating that neither wild-living chimpanzees nor wild-living gorillas are naturally infected with the human parasite. Similarly, epidemiological data indicate that contemporary ape *Laverania* parasites do not normally infect humans (Sundararaman et al. 2013; Délicat-Loembet et al. 2015). Thus, the human parasites have become isolated from, and do not interbreed with, their progenitors infecting gorillas, such that the two are now distinct species and justifying a distinct name (*P. praefalciparum*) for the latter.

The classification established here can serve as a unifying framework for evolutionary and biological studies of members of the *Laverania* subgenus. For example, high-quality *P. gaboni* and *P. reichenowi* genome sequences were recently generated from subpatently infected chimpanzee blood samples using a select whole-genome amplification approach (Sundararaman et al. 2016). These samples were SGA typed at multiple loci to ensure amplification of members of only one, and not multiple, *Laverania* species. Comparison of these and one other full-length *Laverania* genome (Otto et al. 2014) showed that parasites classified as *P. gaboni* and *P. reichenowi* based on subgenomic regions indeed represented distinct species, with no evidence of cross-species mating. Given recent advances in selective amplification and nextgen sequencing approaches (Otto et al. 2014; Sundararaman et al. 2016), it is likely that whole genome sequences from members of all *Laverania* species will be generated long before species-specific morphological data or validated type specimens can be derived.

## Materials and Methods

### Ape Samples

Blood samples were obtained from sanctuary chimpanzees (*P. t. ellioti* and *P. t. troglodytes*) living in outside enclosures in close proximity to wild apes at the Sanaga Yong Wildlife Rescue Center in Cameroon (*n* = 30) and the Tchimpounga Chimpanzee Rehabilitation Center in the Republic of the Congo (*n* = 1) (supplementary table S1, Supplementary Material online). Blood samples were obtained for veterinary purposes only or represented leftover material from yearly health examinations. Most blood samples were preserved in RNAlater (1:1 vol/vol) or as dried blood spots on filter paper without further processing, except for eight samples, which were subjected to density gradient centrifugation in the field to enrich for red blood cells (supplementary table S2, Supplementary Material online). Small quantities of blood were also obtained from one western lowland gorilla (*G.g. gorilla*) of unknown geographic origin (SA), whose body was confiscated by the anti-poaching program of the Cameroonian Ministry of Environment and Forestry. DNA was extracted from whole blood and dried blood spots using the QIAamp Blood DNA mini Kit (Qiagen, Valencia, CA). Ape fecal samples (*n* = 216) were selected from an existing bank of chimpanzee and western gorilla specimens (Keele et al. 2006; Neel et al. 2010; Li et al. 2012; D'arc et al. 2015). These specimens were collected from nonhabituated apes living in remote forest areas, with a two-letter code indicating their field site of origin (fig. 1). Fecal DNA was extracted using the QIAamp Stool DNA mini kit (Qiagen, Valencia, CA). Sample collection was approved by the Ministry of Environment and Forestry in Cameroon and by the Ministry of Forest Economy and Sustainable Development in the Republic of Congo. All samples were shipped in compliance with Convention on International Trade in Endangered Species of Wild Fauna and Flora regulations and country specific import and export permits.

### Single Template Amplification of mtDNA, Apicoplast, and Nuclear Gene Fragments

To derive *Plasmodium* parasite sequences devoid of PCR errors, including *Taq* polymerase-induced misincorporation and template switching, *Laverania* parasite-positive blood and fecal DNA were end point diluted such that fewer than 30% of PCR reactions yielded an amplification product (Liu et al. 2010b). According to a Poisson distribution, a well yielding a PCR product at this dilution will contain only a single amplifiable template more than 83% of the time. Multiple different gene regions were amplified, including the mtDNA *cytB* (956bp), the apicoplast *clpM* gene (390bp), and the nuclear genes *eba*165 (790 bp), *eba*175 (397 bp), *p47* (800 bp), and *ldh* (772 bp). Primers and PCR conditions for *cytB*, *clpM*, *eba*165, *eba*175, and *ldh* have previously been reported (Liu et al. 2010a; Wanaguru et al. 2013; Sundararaman et al. 2016). The *p*47 fragment was amplified using Pfs47F449 (5’-GTAGATGTGATAATAGTAAAACGG-3’) (Anthony et al. 2007) and Pfs47R1 (5’-AATGTATTGGAAAACATTCCATATAC-3’) in the first round, and Pfs47D2F1 (5’-TATCCCAGGACAAGATAAAATAT-3’) and Pfs47R3 (5’- CAAGTTCATTCATATGYTAAMATACAT-3’) in the second round of PCR. For the first round, 2.5 μl of end-point diluted sample DNA was used in a 25 μl reaction volume, containing 0.5 μl dNTPs (10 mM of each dNTP), 10 pmol of each first round primer, 2.5 μl PCR buffer, 0.1 μl BSA solution (50 μg/ml), and 0.25 μl expand long template enzyme mix (Expand Long Template PCR System, Roche). Cycling conditions included an initial denature step of 2 min at 94 °C, followed by 15 cycles of denaturation (94 °C, 10 s), annealing (45 °C, 30 s), and elongation (68 °C, 1 min), followed by 35 cycles of denaturation (94 °C, 10 s), annealing (48 °C, 30 s), and elongation (68 °C, 1 min; with 10-s increments for each successive cycle), followed by a final elongation step of 10 min at 68 °C. For the second round PCR, 2 μl of the first round product was used in 25 μl reaction volume. Cycling conditions included an initial denature step of 2 min at 94 °C, followed by 60 cycles of denaturation (94 °C, 10 s), annealing (52 °C, 30 s), and elongation (68 °C, 1 min), followed by a final elongation step of 10 min at 68 °C. Amplification products were sequenced directly without interim cloning and analyzed using Sequencher (Gene Codes Corporation, Ann Arbor, MI). Sequences containing double peaks in the chromatogram, indicative of the presence of multiple templates or early PCR errors, were discarded. GenBank accession numbers of newly derived SGA sequences are listed in supplementary table S5, Supplementary Material online.

### Phylogenetic Analyses

Sequence alignments were constructed using CLUSTAL W version 2.1 (Larkin et al. 2007) and manually adjusted using MacClade (Maddison and Maddison 1989). Regions that could not be unambiguously aligned were omitted from subsequent phylogenetic analyses. Nuclear gene sequences were subjected to recombination analysis using GARD (Kosakovsky Pond et al. 2006). Evolutionary models for phylogenetic analyses were determined using the Akaike information criterion with jModeltest (version 2.1.4) (Darriba et al. 2012) and PhyML (Guindon and Gascuel 2003). Maximum-likelihood phylogenies with bootstrap support (1,000 replicates) were estimated either jointly with model parameter values by means of PhyML using both nearest-neighbor interchange (NNI) and subtree pruning and regrafting (SPR) with Neighbor Joining and 10 random-addition starting trees (Guindon et al. 2010) or RAxML (Stamatakis 2014). Trees were constructed from partial sequences of the *cytB* gene from the mitochondrial genome (956 bp), *clpM* gene from the apicoplast genome (390 bp), and the *ldh* (772 bp), *eba175* (397 bp), *eba165* (790 bp), and *p47* (800 bp) genes from the nuclear genome.

## Data Availability

Limiting dilution PCR-derived sequences have been deposited in the GenBank nucleotide database under accession codes KT824281, KT824283–KT824285, KT824287, KT824295, KT824296, KT824305, KT824313–KT824315, KT824317, KT824318, KT824320, KT824321, KT824324, KT824335, KT824336, KT824339, KT824340, KT824343, KT824345–KT824348, KT824351, KT824353, KT824354, KT824361–KT824364, KT824366–KT824372, and KU665647–KU665803.

## Supplementary Material

Supplementary figures S1–S6 and tables S1–S5 are available at *Genome Biology and Evolution* online (http://www.gbe.oxfordjournals.org/).

Supplementary Data
