# Supplementary material for: Multigenomic Delineation of Plasmodium Species of the Laverania Subgenus Infecting Wild-Living Chimpanzees and Gorillas
Source: Genome Biol Evol. 2016 Jun 11;8(6):1929–39. doi: 10.1093/gbe/evw128 (PMC4943199; doi:10.1093/gbe/evw128)
Supplement: Supplementary Data [file supp_8_6_1929__index.html]

Multigenomic Delineation of Plasmodium Species of the Laverania Subgenus Infecting Wild-Living Chimpanzees and Gorillas — Supplementary Data 

# Multigenomic Delineation of *Plasmodium* Species of the *Laverania* Subgenus Infecting Wild-Living Chimpanzees and Gorillas

## Supplementary Data

files

- Supplementary Data - pdf file
