# Supplementary material for: Multigenomic Delineation of Plasmodium Species of the Laverania Subgenus Infecting Wild-Living Chimpanzees and Gorillas
Source: Genome Biol Evol. 2016 Jun 11;8(6):1929–39. doi: 10.1093/gbe/evw128 (PMC4943199; doi:10.1093/gbe/evw128)
Supplement: Supplementary Data [file supp_evw128_Supplementary_Info_5-19-16.pdf]

Supplementary Information for *"*Multigenomic Delineation of *Plasmodium* Species of the *Laverania* Subgenus Infecting Wild-living Chimpanzees and Gorillas"

Supplementary Information includes:

Supplementary figures S1-S6

Supplementary tables S1-S5

Supplementary References

Fig. S1. Maximum likelihood phylogeny of *Laverania* mitochondrial DNA sequences. 709 SGA derived *cytB* sequences (956 bp) from 30 blood and 299 fecal samples of sanctuary and wild-living apes are shown, including identical sequences from different samples collected at the same field site (identical sequences from the same sample are excluded). In each panel, the complete phylogenetic tree is shown on the left, with individual *Laverania* clades (panels a-f) magnified on the right. Sequences are color-coded, with capital letters indicating the field site (fig. 1) and lower case letters denoting the species and subspecies origin (ptt: *P. t. troglodytes,* red; pte: *P. t. ellioti,* orange; pts: *P. t. schweinfurthii,* blue; ggg: *G. g. gorilla,* green). SGA dilution and well numbers are also indicated (e.g., GTptt722_160.5 represents an SGA derived sequence amplified from a 1:160 dilution of GTptt722 fecal DNA and identified at position 5 in a plate of multiple PCR reactions). *P. falciparum* and *P. reichenowi* reference sequences are shown in black; asterisks indicate sequences newly generated for this study (for GenBank accession numbers see supplementary table S5, Supplementary Material online). The phylogenetic tree was inferred using RAxML. Bootstrap values (out of 100) are shown for major nodes only (the scale bar represents 0.01 substitutions per site).

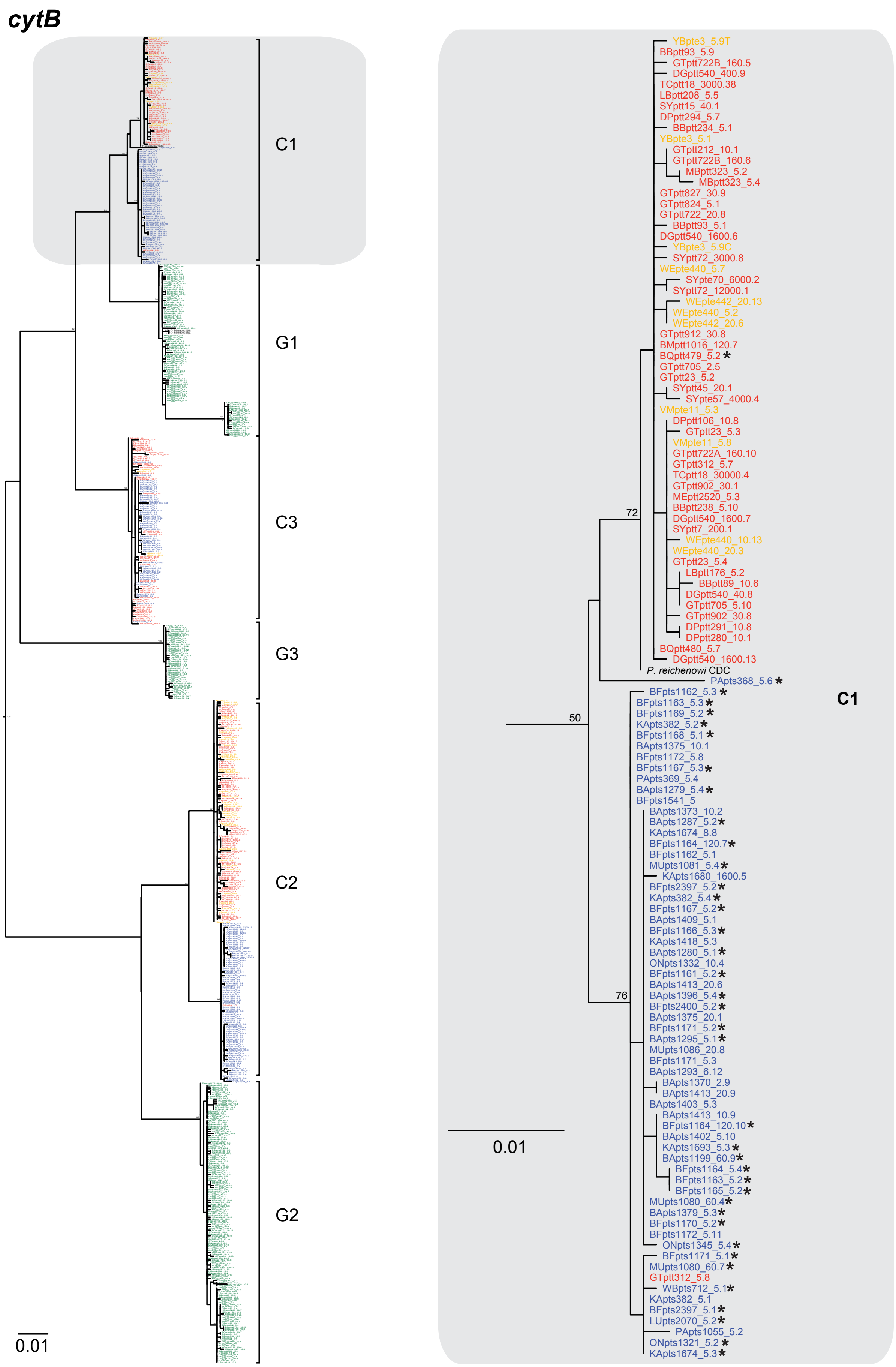
cytB
C1
G1
C3
G3
C2
G2
0.01
YBpte3_5.9T
BBptt93_5.9
GTptt722B_160.5
DGptt540_400.9
TCptt18_3000.38
LBptt208_5.5
SYptt15_40.1
DPptt294_5.7
BBptt234_5.1
YBpte3_5.1
GTptt212_10.1
GTptt722B_160.6
MBptt323_5.2
MBptt323_5.4
GTptt827_30.9
GTptt824_5.1
GTptt722_20.8
BBptt93_5.1
DGptt540_1600.6
YBpte3_5.9C
SYptt72_3000.8
WEpte440_5.7
SYpte70_6000.2
SYptt72_12000.1
WEpte442_20.13
WEpte440_5.2
WEpte442_20.6
GTptt912_30.8
BMptt1016_120.7
BQptt479_5.2 *
GTptt705_2.5
GTptt23_5.2
SYptt45_20.1
SYpte57_4000.4
VMpte11_5.3
DPptt106_10.8
GTptt23_5.3
VMpte11_5.8
GTptt722A_160.10
GTptt312_5.7
TCptt18_30000.4
GTptt902_30.1
MEptt2520_5.3
BBptt238_5.10
DGptt540_1600.7
SYptt7_200.1
WEpte440_10.13
WEpte440_20.3
GTptt23_5.4
LBptt176_5.2
BBptt89_10.6
DGptt540_40.8
GTptt705_5.10
GTptt902_30.8
DPptt291_10.8
DPptt280_10.1
BQptt480_5.7
DGptt540_1600.13
P. reichenowi CDC
PApts368_5.6 *
BFpts1162_5.3 *
BFpts1163_5.3 *
BFpts1169_5.2 *
KApts382_5.2 *
BFpts1168_5.1 *
BApts1375_10.1
BFpts1172_5.8
BFpts1167_5.3 *
PApts369_5.4
BApts1279_5.4 *
BFpts1541_5
BApts1373_10.2
BApts1287_5.2 *
KApts1674_8.8
BFpts1164_120.7 *
BFpts1162_5.1
MUpts1081_5.4 *
KApts1680_1600.5
BFpts2397_5.2 *
KApts382_5.4 *
BFpts1167_5.2 *
BApts1409_5.1
BFpts1166_5.3 *
KApts1418_5.3
BApts1280_5.1 *
ONpts1332_10.4
BFpts1161_5.2 *
BApts1413_20.6
BApts1396_5.4 *
BFpts2400_5.2 *
BApts1375_20.1
BFpts1171_5.2 *
BApts1295_5.1 *
MUpts1086_20.8
BFpts1171_5.3
BApts1293_6.12
BApts1370_2.9
BApts1413_20.9
BApts1403_5.3
BApts1413_10.9
BFpts1164_120.10 *
BApts1402_5.10
KApts1693_5.3 *
BApts1199_60.9 *
BFpts1164_5.4 *
BFpts1163_5.2 *
BFpts1165_5.2 *
MUpts1080_60.4 *
BApts1379_5.3 *
BFpts1170_5.2 *
BFpts1172_5.11
ONpts1345_5.4 *
BFpts1171_5.1 *
MUpts1080_60.7 *
GTptt312_5.8
WBpts712_5.1 *
KApts382_5.1
BFpts2397_5.1 *
LUpts2070_5.2 *
PApts1055_5.2
ONpts1321_5.2 *
KApts1674_5.3 *
72
50
76
C1
0.01

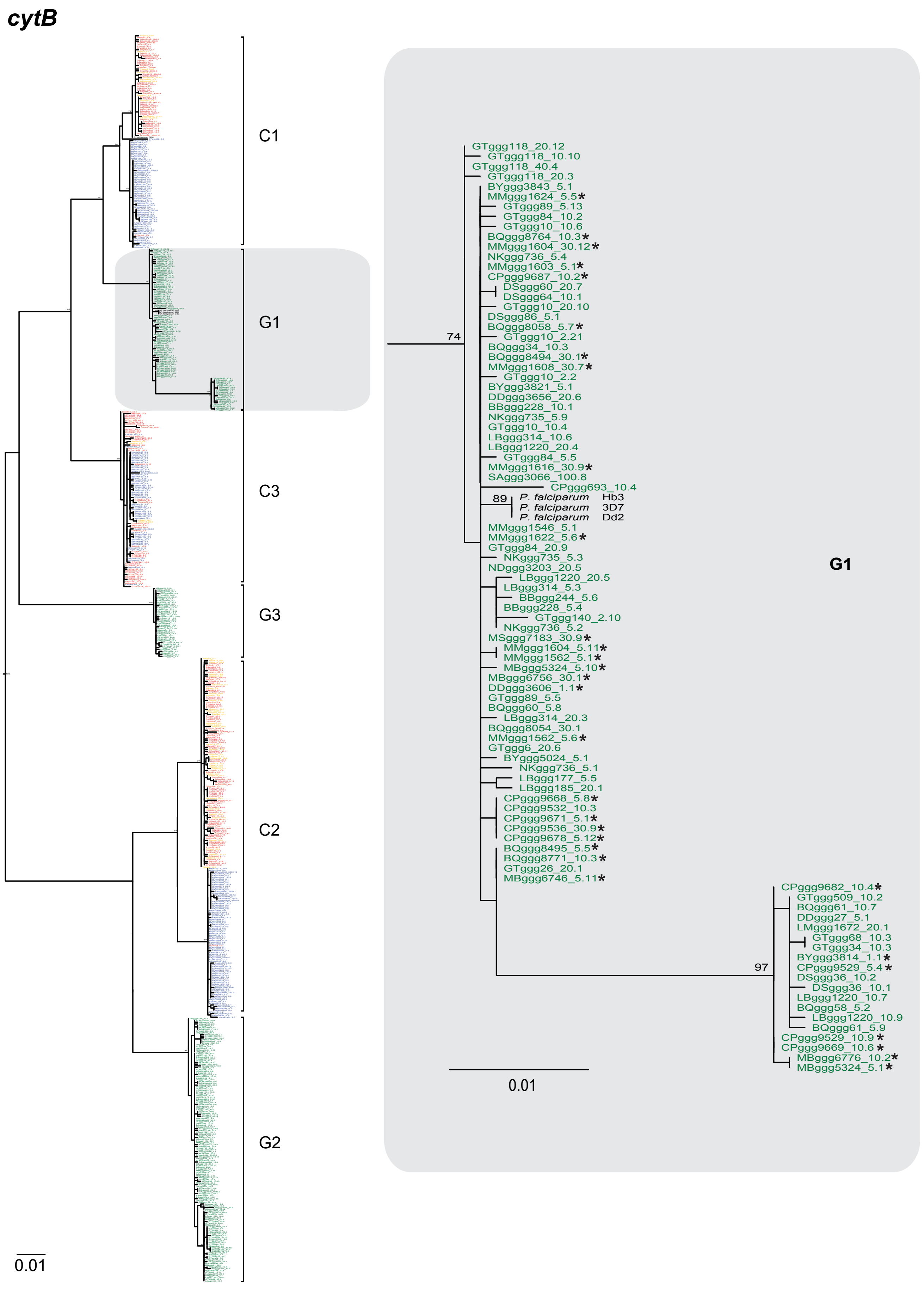
cytB
C1
G1
C3
G3
C2
G2
0.01
74
GTggg118_20.12
GTggg118_10.10
GTggg118_40.4
GTggg118_20.3
BYggg3843_5.1
MMggg1624_5.5*
GTggg89_5.13
GTggg84_10.2
GTggg10_10.6
BQggg8764_10.3*
MMggg1604_30.12*
NKggg736_5.4
MMggg1603_5.1*
CPggg9687_10.2*
DSggg60_20.7
DSggg64_10.1
GTggg10_20.10
DSggg86_5.1
BQggg8058_5.7*
GTggg10_2.21
BQggg34_10.3
BQggg8494_30.1*
MMggg1608_30.7*
GTggg10_2.2
BYggg3821_5.1
DDggg3656_20.6
BBggg228_10.1
NKggg735_5.9
GTggg10_10.4
LBggg314_10.6
LBggg1220_20.4
GTggg84_5.5
MMggg1616_30.9*
SAggg3066_100.8
CPggg693_10.4
89
P. falciparum Hb3
P. falciparum 3D7
P. falciparum Dd2
MMggg1546_5.1
MMggg1622_5.6*
GTggg84_20.9
NKggg735_5.3
NDggg3203_20.5
LBggg1220_20.5
LBggg314_5.3
BBggg244_5.6
BBggg228_5.4
GTggg140_2.10
NKggg736_5.2
MSggg7183_30.9*
MMggg1604_5.11*
MMggg1562_5.1*
MBggg5324_5.10*
MBggg6756_30.1*
DDggg3606_1.1*
GTggg89_5.5
BQggg60_5.8
LBggg314_20.3
BQggg8054_30.1
MMggg1562_5.6*
GTggg6_20.6
BYggg5024_5.1
NKggg736_5.1
LBggg177_5.5
LBggg185_20.1
CPggg9668_5.8*
CPggg9532_10.3
CPggg9671_5.1*
CPggg9536_30.9*
CPggg9678_5.12*
BQggg8495_5.5*
BQggg8771_10.3*
GTggg26_20.1
MBggg6746_5.11*
G1
97
CPggg9682_10.4*
GTggg509_10.2
BQggg61_10.7
DDggg27_5.1
LMggg1672_20.1
GTggg68_10.3
GTggg34_10.3
BYggg3814_1.1*
CPggg9529_5.4*
DSggg36_10.2
DSggg36_10.1
LBggg1220_10.7
BQggg58_5.2
LBggg1220_10.9
BQggg61_5.9
CPggg9529_10.9*
CPggg9669_10.6*
MBggg6776_10.2*
MBggg5324_5.1*
0.01

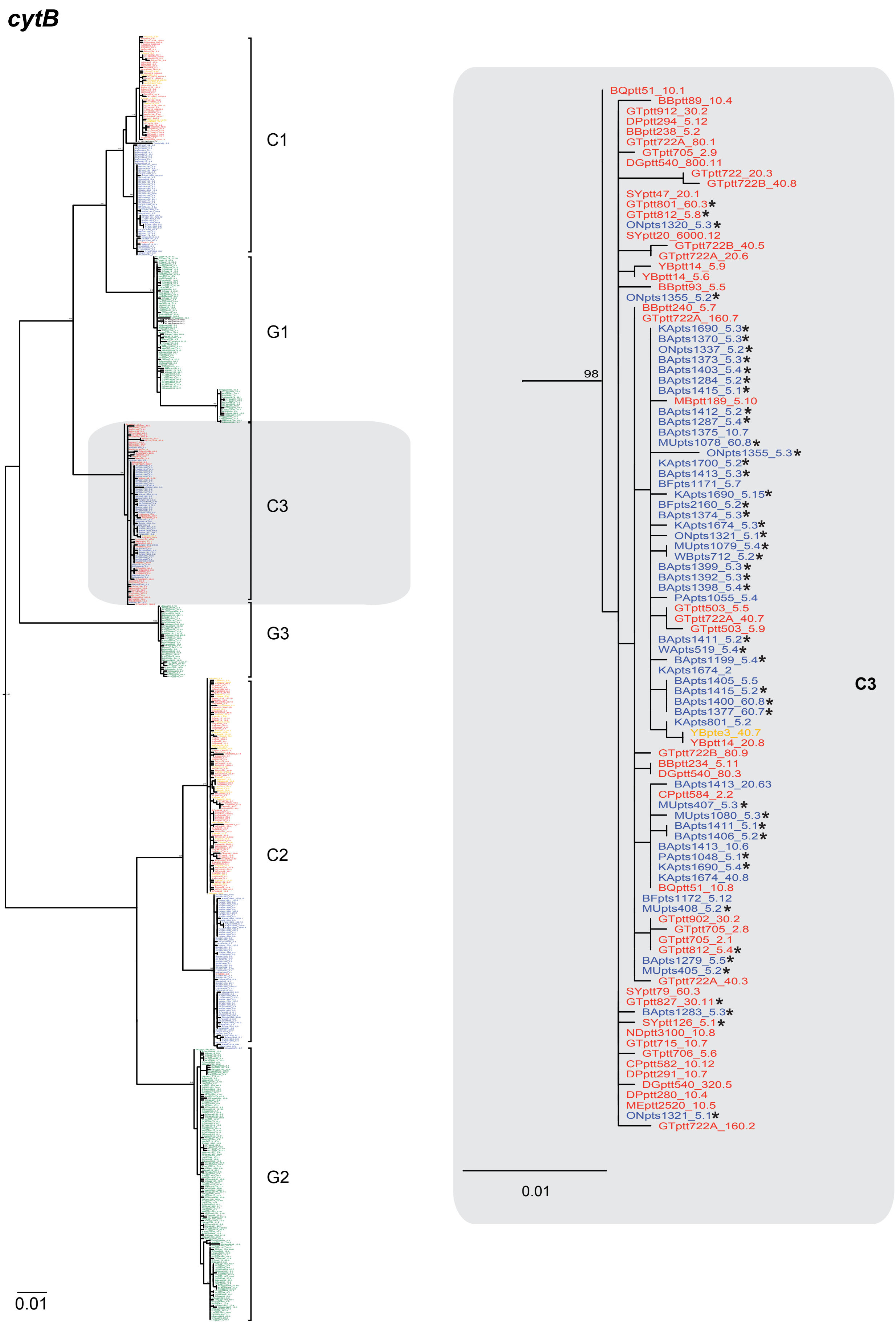
cytB
C1
G1
C3
G3
C2
G2
0.01
BQptt51_10.1
BBptt89_10.4
GTptt912_30.2
DPptt294_5.12
BBptt238_5.2
GTptt722A_80.1
GTptt705_2.9
DGptt540_800.11
GTptt722_20.3
GTptt722B_40.8
SYptt47_20.1
GTptt801_60.3*
GTptt812_5.8*
ONpts1320_5.3*
SYptt20_6000.12
GTptt722B_40.5
GTptt722A_20.6
YBptt14_5.9
YBptt14_5.6
BBptt93_5.5
ONpts1355_5.2*
BBptt240_5.7
GTptt722A_160.7
KApts1690_5.3*
BApts1370_5.3*
ONpts1337_5.2*
BApts1373_5.3*
BApts1403_5.4*
BApts1284_5.2*
BApts1415_5.1*
MBptt189_5.10
BApts1412_5.2*
BApts1287_5.4*
BApts1375_10.7
MUpts1078_60.8*
ONpts1355_5.3*
KApts1700_5.2*
BApts1413_5.3*
BFpts1171_5.7
KApts1690_5.15*
BFpts2160_5.2*
BApts1374_5.3*
KApts1674_5.3*
ONpts1321_5.1*
MUpts1079_5.4*
WBpts712_5.2*
BApts1399_5.3*
BApts1392_5.3*
BApts1398_5.4*
PApts1055_5.4
GTptt503_5.5
GTptt722A_40.7
GTptt503_5.9
BApts1411_5.2*
WApts519_5.4*
BApts1199_5.4*
KApts1674_2
BApts1405_5.5
BApts1415_5.2*
BApts1400_60.8*
BApts1377_60.7*
KApts801_5.2
YBpte3_40.7
YBptt14_20.8
GTptt722B_80.9
BBptt234_5.11
DGptt540_80.3
BApts1413_20.63
CPptt584_2.2
MUpts407_5.3*
MUpts1080_5.3*
BApts1411_5.1*
BApts1406_5.2*
BApts1413_10.6
PApts1048_5.1*
KApts1690_5.4*
KApts1674_40.8
BQptt51_10.8
BFpts1172_5.12
MUpts408_5.2*
GTptt902_30.2
GTptt705_2.8
GTptt705_2.1
GTptt812_5.4*
BApts1279_5.5*
MUpts405_5.2*
GTptt722A_40.3
SYptt79_60.3
GTptt827_30.11*
BApts1283_5.3*
SYptt126_5.1*
NDptt3100_10.8
GTptt715_10.7
GTptt706_5.6
CPptt582_10.12
DPptt291_10.7
DGptt540_320.5
DPptt280_10.4
MEptt2520_10.5
ONpts1321_5.1*
GTptt722A_160.2
98
C3
0.01

cytB

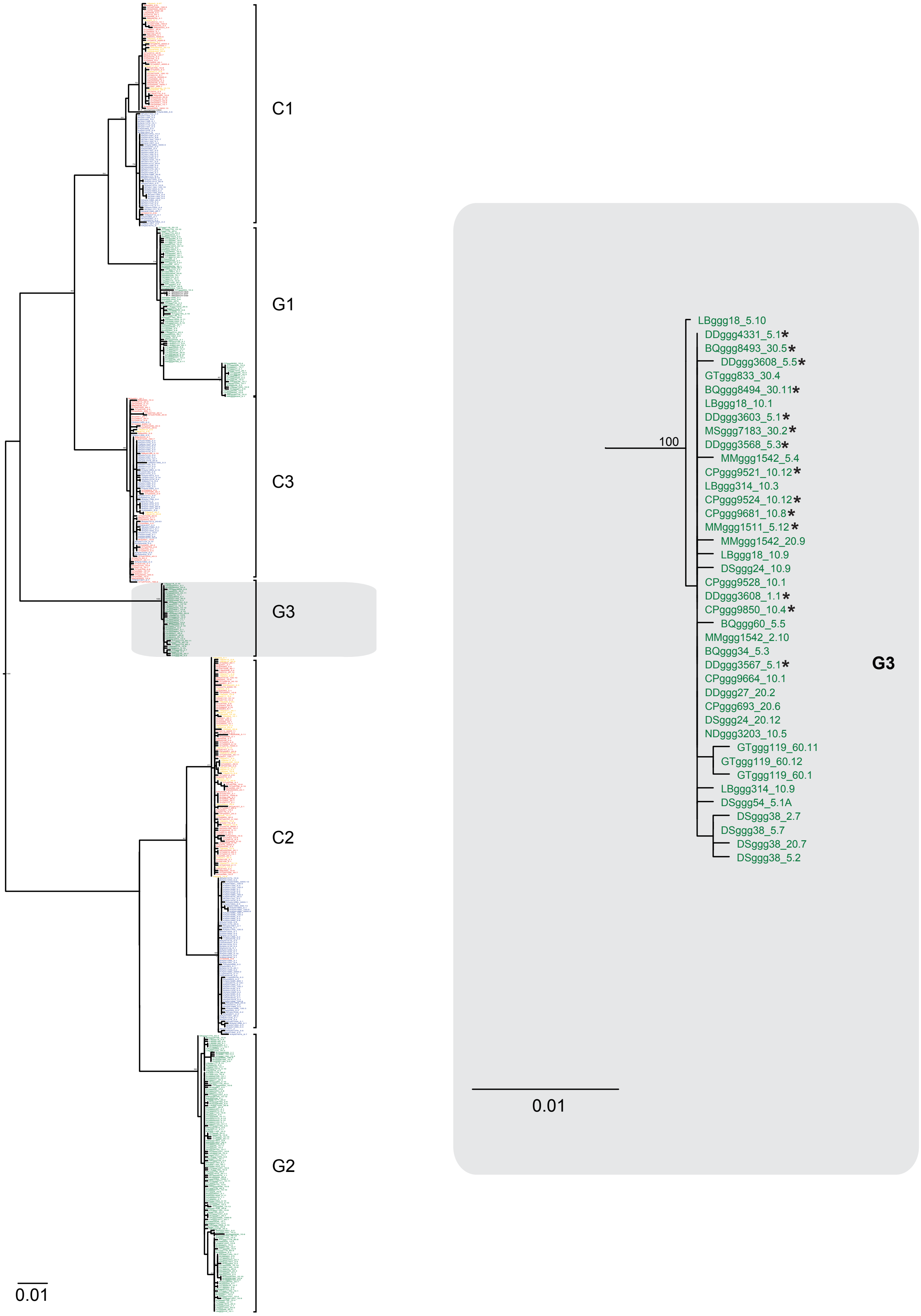
C1
G1
C3
G3
C2
G2
0.01
LBggg18_5.10
DDggg4331_5.1*
BQggg8493_30.5*
DDggg3608_5.5*
GTggg833_30.4
BQggg8494_30.11*
LBggg18_10.1
DDggg3603_5.1*
MSggg7183_30.2*
DDggg3568_5.3*
MMggg1542_5.4
CPggg9521_10.12*
LBggg314_10.3
CPggg9524_10.12*
CPggg9681_10.8*
MMggg1511_5.12*
MMggg1542_20.9
LBggg18_10.9
DSggg24_10.9
CPggg9528_10.1
DDggg3608_1.1*
CPggg9850_10.4*
BQggg60_5.5
MMggg1542_2.10
BQggg34_5.3
DDggg3567_5.1*
CPggg9664_10.1
DDggg27_20.2
CPggg693_20.6
DSggg24_20.12
NDggg3203_10.5
GTggg119_60.11
GTggg119_60.12
GTggg119_60.1
LBggg314_10.9
DSggg54_5.1A
DSggg38_2.7
DSggg38_5.7
DSggg38_20.7
DSggg38_5.2
100
0.01
G3

cytB

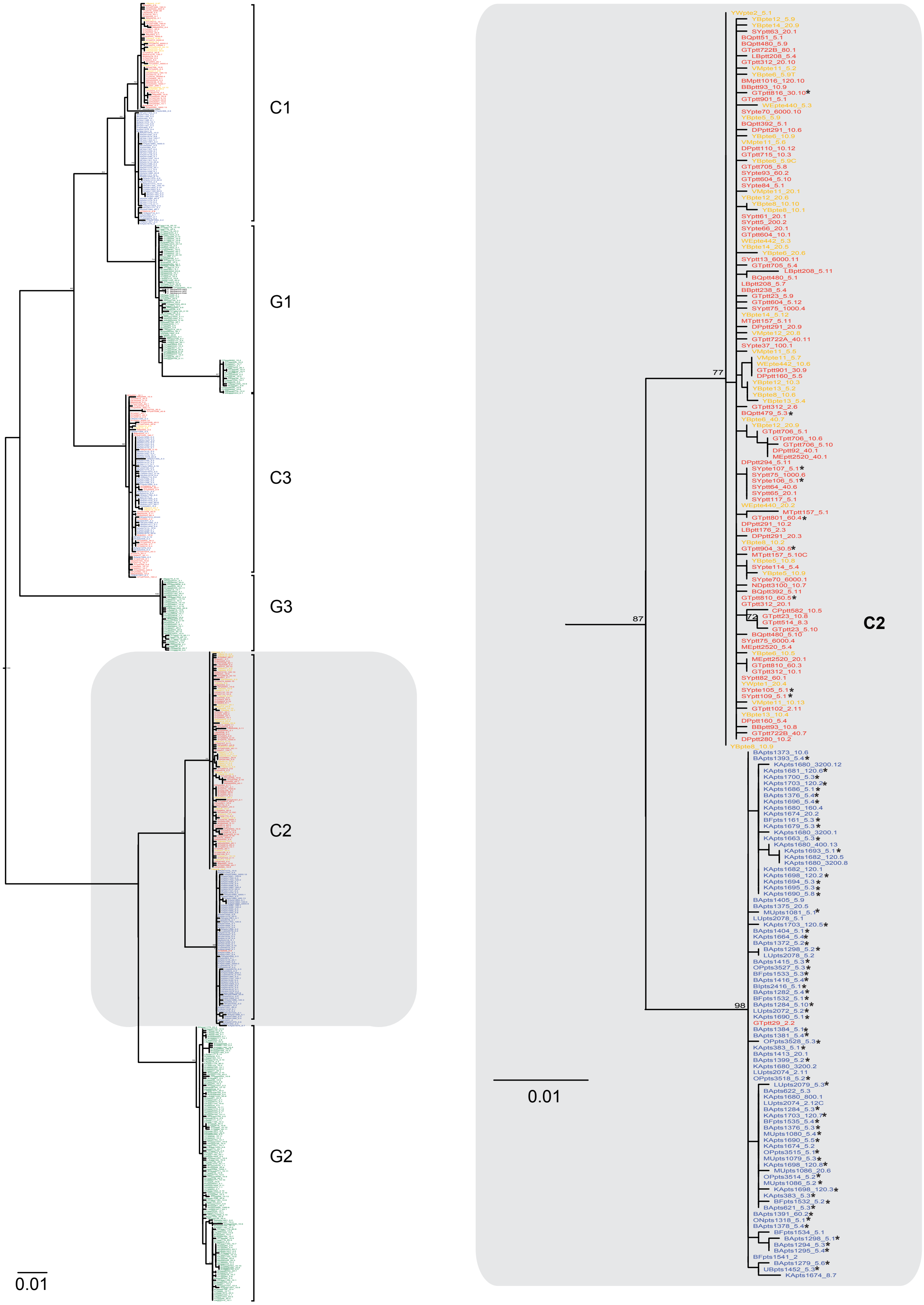

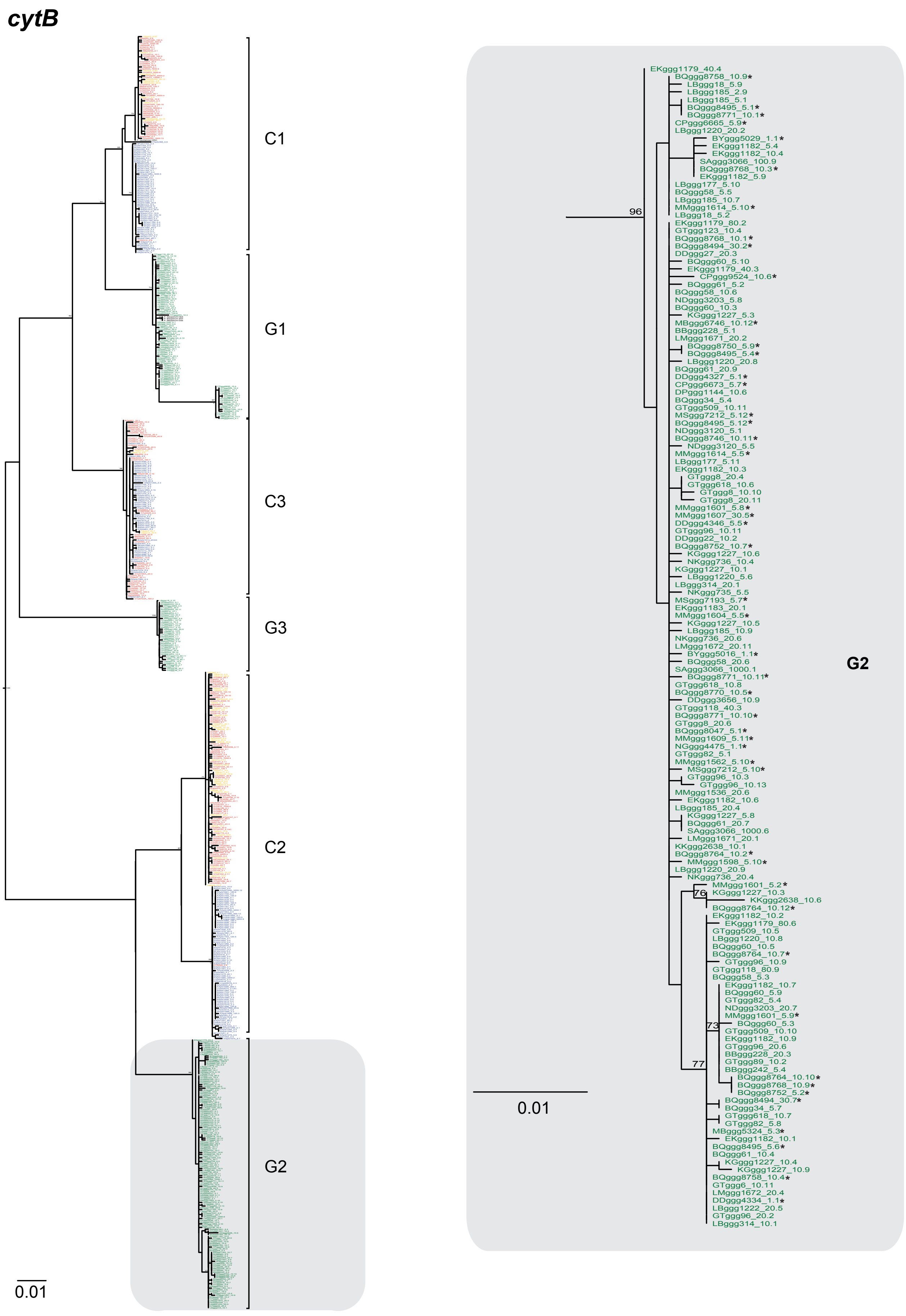

cytB
C1
G1
C3
G3
C2
G2
0.01
EKggg1179_40.4
BQggg8758_10.9*
LBggg18_5.9
LBggg185_2.9
LBggg185_5.1
BQggg8495_5.1*
BQggg8771_10.1*
CPggg6665_5.9*
LBggg1220_20.2
BYggg5029_1.1*
EKggg1182_5.4
EKggg1182_10.4
SAggg3066_100.9
BQggg8768_10.3*
EKggg1182_5.9
LBggg177_5.10
BQggg58_5.5
LBggg185_10.7
MMggg1614_5.10*
LBggg18_5.2
96
EKggg1179_80.2
GTggg123_10.4
BQggg8768_10.1*
BQggg8494_30.2*
DDggg27_20.3
BQggg60_5.10
EKggg1179_40.3
CPggg9524_10.6*
BQggg61_5.2
BQggg58_10.6
NDggg3203_5.8
BQggg60_10.3
KGggg1227_5.3
MBggg6746_10.12*
BBggg228_5.1
LMggg1671_20.2
BQggg8750_5.9*
BQggg8495_5.4*
LBggg1220_20.8
BQggg61_20.9
DDggg4327_5.1*
CPggg6673_5.7*
DPggg1144_10.6
BQggg34_5.4
GTggg509_10.11
MSggg7212_5.12*
BQggg8495_5.12*
NDggg3120_5.1
BQggg8746_10.11*
NDggg3120_5.5
MMggg1614_5.5*
LBggg177_5.11
EKggg1182_10.3
GTggg8_20.4
GTggg618_10.6
GTggg8_10.10
GTggg8_20.11
MMggg1601_5.8*
MMggg1607_30.5*
DDggg4346_5.5*
GTggg96_10.11
DDggg22_10.2
BQggg8752_10.7*
KGggg1227_10.6
NKggg736_10.4
KGggg1227_10.1
LBggg1220_5.6
LBggg314_20.1
NKggg735_5.5
MSggg7193_5.7*
EKggg1183_20.1
MMggg1604_5.5*
KGggg1227_10.5
LBggg185_10.9
NKggg736_20.6
LMggg1672_20.11
BYggg5016_1.1*
BQggg58_20.6
SAggg3066_1000.1
BQggg8771_10.11*
GTggg618_10.8
BQggg8770_10.5*
DDggg3656_10.9
GTggg118_40.3
BQggg8771_10.10*
GTggg8_20.6
BQggg8047_5.1*
MMggg1609_5.11*
NGggg4475_1.1*
GTggg82_5.1
MMggg1562_5.10*
MSggg7212_5.10*
GTggg96_10.3
GTggg96_10.13
MMggg1536_20.6
EKggg1182_10.6
LBggg185_20.4
KGggg1227_5.8
BQggg61_20.7
SAggg3066_1000.6
LMggg1671_20.1
KKggg2638_10.1
BQggg8764_10.2*
MMggg1598_5.10*
LBggg1220_20.9
NKggg736_20.4
MMggg1601_5.2*
76
KGggg1227_10.3
KKggg2638_10.6
BQggg8764_10.12*
EKggg1182_10.2
EKggg1179_80.6
GTggg509_10.5
LBggg1220_10.8
BQggg60_10.5
BQggg8764_10.7*
GTggg96_10.9
GTggg118_80.9
BQggg58_5.3
EKggg1182_10.7
BQggg60_5.9
GTggg82_5.4
NDggg3203_20.7
MMggg1601_5.9*
73
BQggg60_5.3
GTggg509_10.10
EKggg1182_10.9
GTggg96_20.6
BBggg228_20.3
GTggg89_10.2
77
BBggg242_5.4
BQggg8764_10.10*
BQggg8768_10.9*
BQggg8752_5.2*
BQggg8494_30.7*
BQggg34_5.7
GTggg618_10.7
GTggg82_5.8
MBggg5324_5.3*
EKggg1182_10.1
BQggg8495_5.6*
BQggg61_10.4
KGggg1227_10.4
KGggg1227_10.9
BQggg8758_10.4*
GTggg6_10.11
LMggg1672_20.4
DDggg4334_1.1*
LBggg1222_20.5
GTggg96_20.2
LBggg314_10.1
0.01

Fig. S2. Maximum likelihood phylogeny of *Laverania* lactate dehydrogenase (*ldh*) gene sequences. 85 SGA-derived *ldh* sequences (772 bp) from 22 blood and 31 fecal samples of sanctuary and wild-living apes are shown, including identical sequences from different samples collected at the same field site (identical sequences from the same sample are excluded). Sequences are labeled and color-coded as in supplementary fig. S1. Asterisks indicate sequences newly generated for this study (for GenBank accession numbers see supplementary table S5, Supplementary Material online). The phylogenetic tree was inferred using PhyML. Bootstrap values (out of 1,000) are shown for major nodes only (the scale bar represents 0.01 substitutions per site).

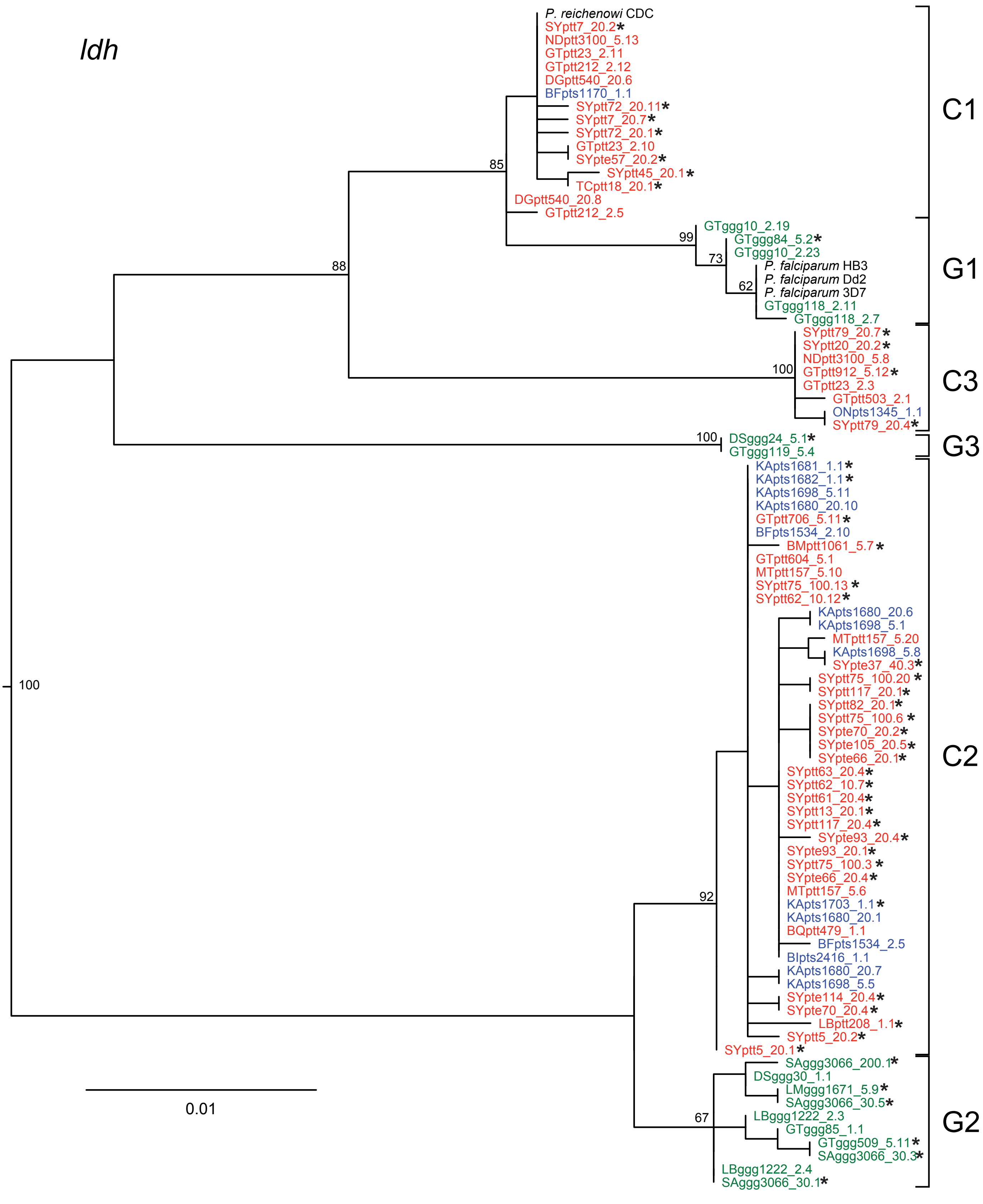

ldh
P. reichenowi CDC
SYptt7_20.2*
NDptt3100_5.13
GTptt23_2.11
GTptt212_2.12
DGptt540_20.6
BFpts1170_1.1
SYptt72_20.11*
SYptt7_20.7*
SYptt72_20.1*
GTptt23_2.10
SYpte57_20.2*
SYptt45_20.1*
TCptt18_20.1*
DGptt540_20.8
GTptt212_2.5
GTggg10_2.19
GTggg84_5.2*
GTggg10_2.23
P. falciparum HB3
P. falciparum Dd2
P. falciparum 3D7
GTggg118_2.11
GTggg118_2.7
SYptt79_20.7*
SYptt20_20.2*
NDptt3100_5.8
GTptt912_5.12*
GTptt23_2.3
GTptt503_2.1
ONpts1345_1.1
SYptt79_20.4*
DSggg24_5.1*
GTggg119_5.4
KApts1681_1.1*
KApts1682_1.1*
KApts1698_5.11
KApts1680_20.10
GTptt706_5.11*
BFpts1534_2.10
BMptt1061_5.7*
GTptt604_5.1
MTptt157_5.10
SYptt75_100.13*
SYptt62_10.12*
KApts1680_20.6
KApts1698_5.1
MTptt157_5.20
KApts1698_5.8
SYpte37_40.3*
SYptt75_100.20*
SYptt117_20.1*
SYptt82_20.1*
SYptt75_100.6*
SYpte70_20.2*
SYpte105_20.5*
SYpte66_20.1*
SYptt63_20.4*
SYptt62_10.7*
SYptt61_20.4*
SYptt13_20.1*
SYptt117_20.4*
SYpte93_20.4*
SYpte93_20.1*
SYptt75_100.3*
SYpte66_20.4*
MTptt157_5.6
KApts1703_1.1*
KApts1680_20.1
BQptt479_1.1
BFpts1534_2.5
BIpts2416_1.1
KApts1680_20.7
KApts1698_5.5
SYpte114_20.4*
SYpte70_20.4*
LBptt208_1.1*
SYptt5_20.2*
SYptt5_20.1*
SAggg3066_200.1*
DSggg30_1.1
LMggg1671_5.9*
SAggg3066_30.5*
LBggg1222_2.3
GTggg85_1.1
GTggg509_5.11*
SAggg3066_30.3*
LBggg1222_2.4
SAggg3066_30.1*
85
88
99
73
62
100
100
100
92
67
C1
G1
C3
G3
C2
G2
0.01

Fig. S3. Maximum likelihood phylogeny of *Laverania* erythrocyte binding antigen 175 (*eba175*) gene sequences. 85 SGA-derived *eba175* sequences (397 bp) from 17 blood and 37 fecal samples of sanctuary and wild-living apes are shown, including identical sequences from different samples collected at the same field site (identical sequences from the same sample are excluded). Sequences are labeled and color-coded as in supplementary fig. S1. Asterisks indicate sequences newly generated for this study (for GenBank accession numbers see supplementary table S5, Supplementary Material online). The phylogenetic tree was inferred using PhyML. Bootstrap values (out of 1,000) are shown for major nodes only (the scale bar represents 0.01 substitutions per site).

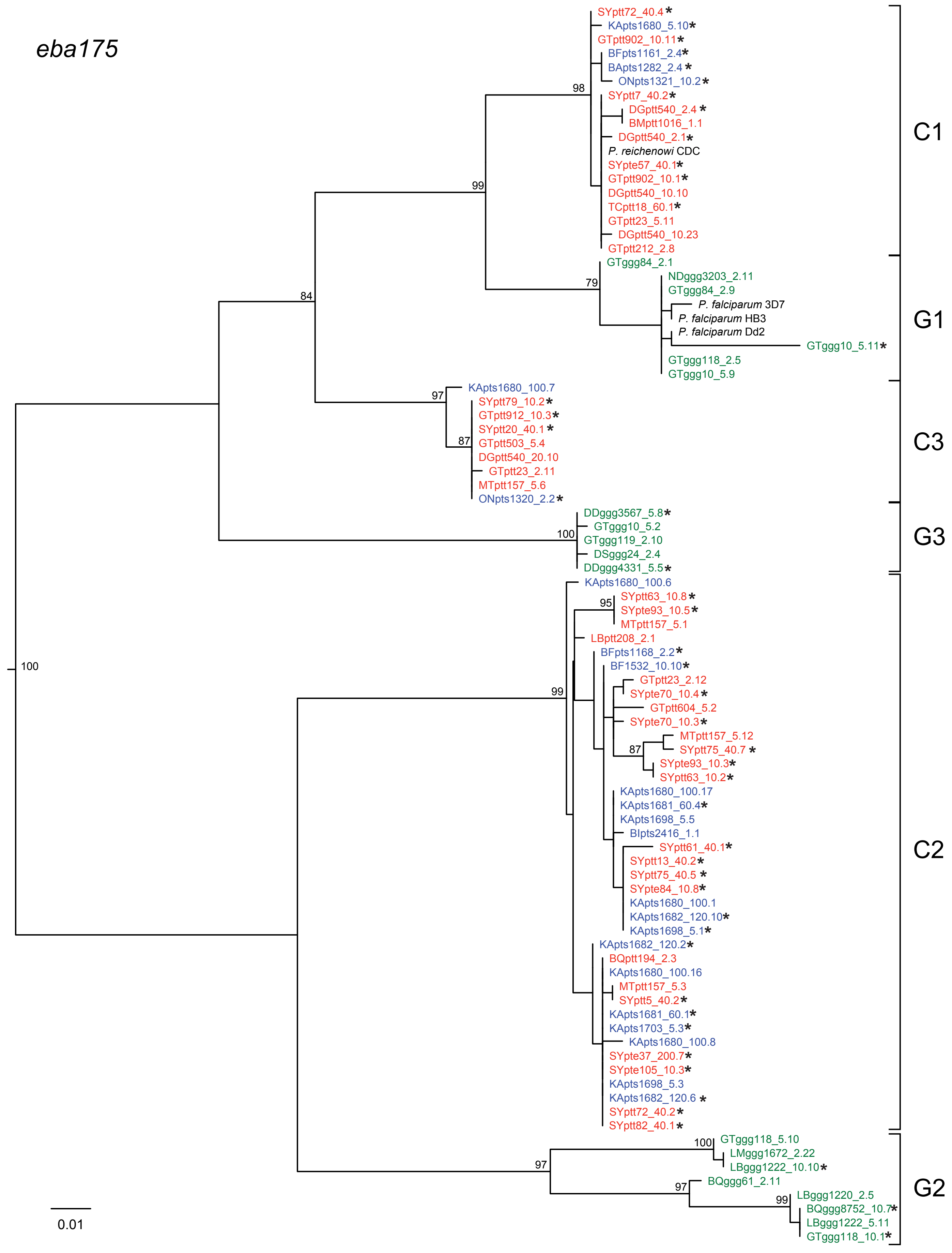
eba175
SYptt72_40.4*
KApts1680_5.10*
GTptt902_10.11*
BFpts1161_2.4*
BApts1282_2.4*
ONpts1321_10.2*
SYptt7_40.2*
DGptt540_2.4*
BMptt1016_1.1
DGptt540_2.1*
P. reichenowi CDC
SYpte57_40.1*
GTptt902_10.1*
DGptt540_10.10
TCptt18_60.1*
GTptt23_5.11
DGptt540_10.23
GTptt212_2.8
GTggg84_2.1
NDggg3203_2.11
GTggg84_2.9
P. falciparum 3D7
P. falciparum HB3
P. falciparum Dd2
GTggg10_5.11*
GTggg118_2.5
GTggg10_5.9
KApts1680_100.7
SYptt79_10.2*
GTptt912_10.3*
SYptt20_40.1*
GTptt503_5.4
DGptt540_20.10
GTptt23_2.11
MTptt157_5.6
ONpts1320_2.2*
DDggg3567_5.8*
GTggg10_5.2
GTggg119_2.10
DSggg24_2.4
DDggg4331_5.5*
KApts1680_100.6
SYptt63_10.8*
SYpte93_10.5*
MTptt157_5.1
LBptt208_2.1
BFpts1168_2.2*
BF1532_10.10*
GTptt23_2.12
SYpte70_10.4*
GTptt604_5.2
SYpte70_10.3*
MTptt157_5.12
SYptt75_40.7*
SYpte93_10.3*
SYptt63_10.2*
KApts1680_100.17
KApts1681_60.4*
KApts1698_5.5
BIpts2416_1.1
SYptt61_40.1*
SYptt13_40.2*
SYptt75_40.5*
SYpte84_10.8*
KApts1680_100.1
KApts1682_120.10*
KApts1698_5.1*
KApts1682_120.2*
BQptt194_2.3
KApts1680_100.16
MTptt157_5.3
SYptt5_40.2*
KApts1681_60.1*
KApts1703_5.3*
KApts1680_100.8
SYpte37_200.7*
SYpte105_10.3*
KApts1698_5.3
KApts1682_120.6*
SYptt72_40.2*
SYptt82_40.1*
GTggg118_5.10
LMggg1672_2.22
LBggg1222_10.10*
BQggg61_2.11
LBggg1220_2.5
BQggg8752_10.7*
LBggg1222_5.11
GTggg118_10.1*
98
99
84
79
97
87
100
100
99
95
87
100
97
97
99
C1
G1
C3
G3
C2
G2
0.01

Fig. S4. Maximum likelihood phylogeny of *Laverania* erythrocyte binding antigen 165 (*eba165*) gene sequences. 101 SGA-derived *eba165* sequences (790 bp) from 23 blood and 49 fecal samples of sanctuary and wild-living apes are shown, including identical sequences from different samples collected at the same field site (identical sequences from the same sample are excluded). Sequences are labeled and color-coded as in supplementary fig. S1. Asterisks indicate sequences newly generated for this study (for GenBank accession numbers see supplementary table S5, Supplementary Material online). The phylogenetic tree was inferred using PhyML. Bootstrap values (out of 1,000) are shown for major nodes only (the scale bar represents 0.01 substitutions per site).

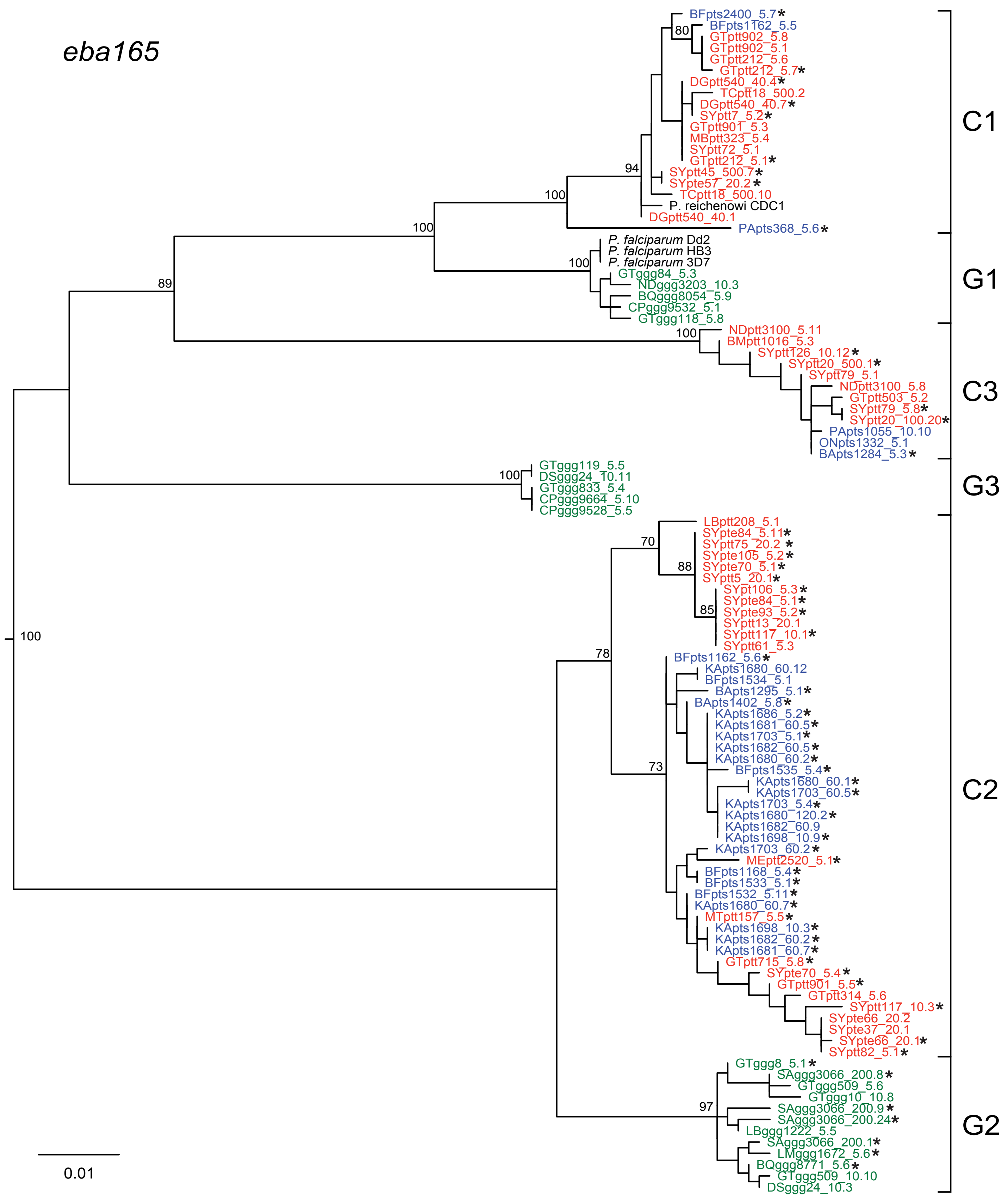
eba165
BFpts2400_5.7*
BFpts1162_5.5
GTptt902_5.8
GTptt902_5.1
GTptt212_5.6
GTptt212_5.7*
DGptt540_40.4*
TCptt18_500.2
DGptt540_40.7*
SYptt7_5.2*
GTptt901_5.3
MBptt323_5.4
SYptt72_5.1
GTptt212_5.1*
SYptt45_500.7*
SYpte57_20.2*
TCptt18_500.10
P. reichenowi CDC1
DGptt540_40.1
PApts368_5.6*
P. falciparum Dd2
P. falciparum HB3
P. falciparum 3D7
GTggg84_5.3
NDggg3203_10.3
BQggg8054_5.9
CPggg9532_5.1
GTggg118_5.8
NDptt3100_5.11
BMptt1016_5.3
SYptt126_10.12*
SYptt20_500.1*
SYptt79_5.1
NDptt3100_5.8
GTptt503_5.2
SYptt79_5.8*
SYptt20_100.20*
PApts1055_10.10
ONpts1332_5.1
BApts1284_5.3*
GTggg119_5.5
DSggg24_10.11
GTggg833_5.4
CPggg9664_5.10
CPggg9528_5.5
LBptt208_5.1
SYpte84_5.11*
SYptt75_20.2*
SYpte105_5.2*
SYpte70_5.1*
SYptt5_20.1*
SYpt106_5.3*
SYpte84_5.1*
SYpte93_5.2*
SYptt13_20.1
SYptt117_10.1*
SYptt61_5.3
BFpts1162_5.6*
KApts1680_60.12
BFpts1534_5.1
BApts1295_5.1*
BApts1402_5.8*
KApts1686_5.2*
KApts1681_60.5*
KApts1703_5.1*
KApts1682_60.5*
KApts1680_60.2*
BFpts1535_5.4*
KApts1680_60.1*
KApts1703_60.5*
KApts1703_5.4*
KApts1680_120.2*
KApts1682_60.9
KApts1698_10.9*
KApts1703_60.2*
MEptt2520_5.1*
BFpts1168_5.4*
BFpts1533_5.1*
BFpts1532_5.11*
KApts1680_60.7*
MTptt157_5.5*
KApts1698_10.3*
KApts1682_60.2*
KApts1681_60.7*
GTptt715_5.8*
SYpte70_5.4*
GTptt901_5.5*
GTptt314_5.6
SYptt117_10.3*
SYpte66_20.2
SYpte37_20.1
SYpte66_20.1*
SYptt82_5.1*
GTggg8_5.1*
SAggg3066_200.8*
GTggg509_5.6
GTggg10_10.8
SAggg3066_200.9*
SAggg3066_200.24*
LBggg1222_5.5
SAggg3066_200.1*
LMggg1672_5.6*
BQggg8771_5.6*
GTggg509_10.10
DSggg24_10.3
80
94
100
100
100
100
89
100
100
100
70
88
85
78
73
97
C1
G1
C3
G3
C2
G2
0.01

Fig. S5. Maximum likelihood phylogeny of *Laverania* gametocyte surface protein P47 (*p47*) gene sequences. 114 SGA-derived *p47* sequences (800 bp) from 26 blood and 37 fecal samples of sanctuary and wild-living apes are shown, including identical sequences from different samples collected at the same field site (identical sequences from the same sample are excluded). Sequences are labeled and color-coded as in supplementary fig. S1. GenBank accession numbers are listed in supplementary table S5 (Supplementary Material online). The phylogenetic tree was inferred using PhyML. Bootstrap values (out of 1,000) are shown for major nodes only (the scale bar represents 0.01 substitutions per site).

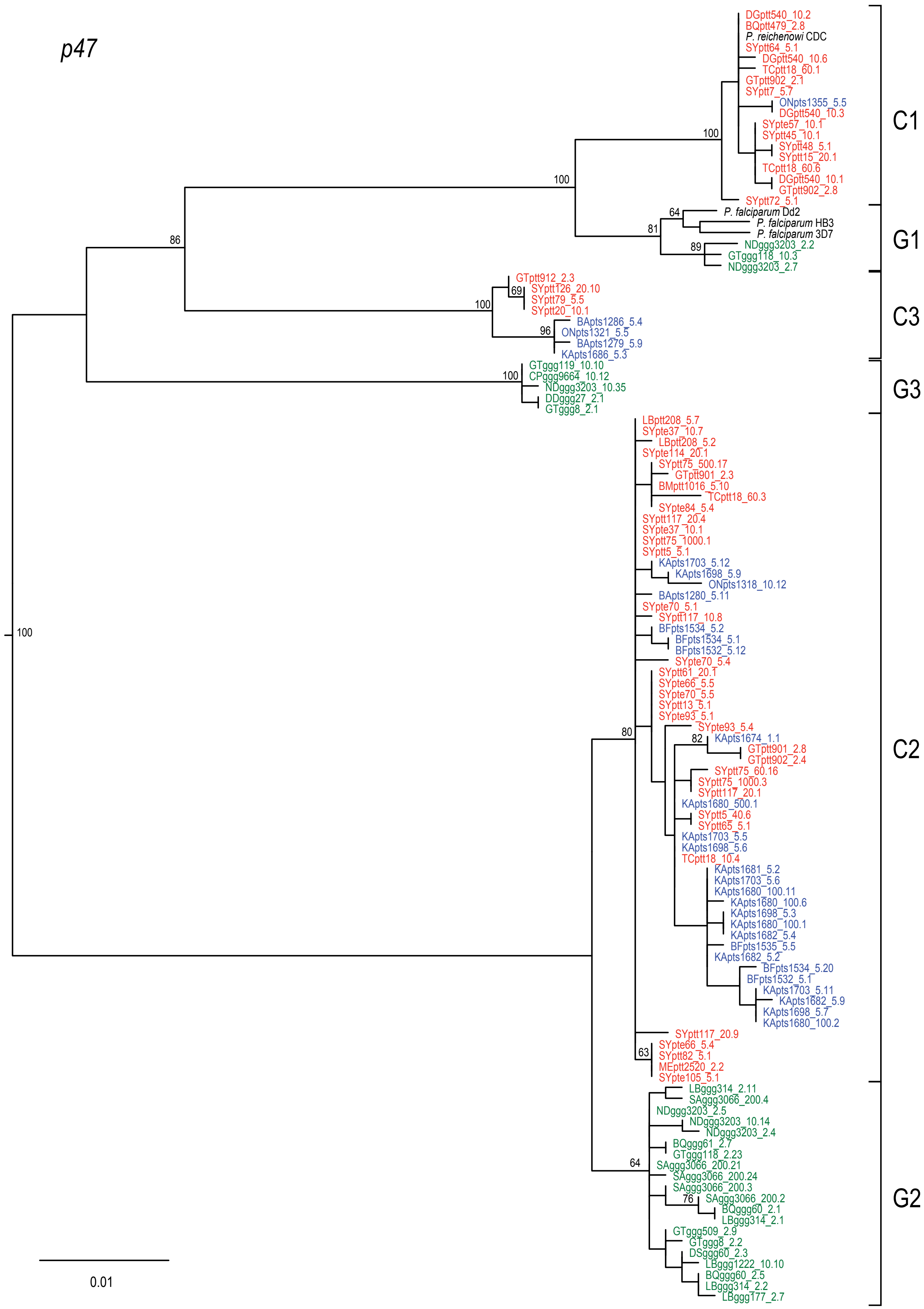
p47
DGptt540_10.2
BQptt479_2.8
P. reichenowi CDC
SYptt64_5.1
DGptt540_10.6
TCptt18_60.1
GTptt902_2.1
SYptt7_5.7
ONpts1355_5.5
DGptt540_10.3
SYpte57_10.1
SYptt45_10.1
SYptt48_5.1
SYptt15_20.1
TCptt18_60.6
DGptt540_10.1
GTptt902_2.8
SYptt72_5.1
P. falciparum Dd2
P. falciparum HB3
P. falciparum 3D7
NDggg3203_2.2
GTggg118_10.3
NDggg3203_2.7
GTptt912_2.3
SYptt126_20.10
SYptt79_5.5
SYptt20_10.1
BApts1286_5.4
ONpts1321_5.5
BApts1279_5.9
KApts1686_5.3
GTggg119_10.10
CPggg9664_10.12
NDggg3203_10.35
DDggg27_2.1
GTggg8_2.1
LBptt208_5.7
SYpte37_10.7
LBptt208_5.2
SYpte114_20.1
SYptt75_500.17
GTptt901_2.3
BMptt1016_5.10
TCptt18_60.3
SYpte84_5.4
SYptt117_20.4
SYpte37_10.1
SYptt75_1000.1
SYptt5_5.1
KApts1703_5.12
KApts1698_5.9
ONpts1318_10.12
BApts1280_5.11
SYpte70_5.1
SYptt117_10.8
BFpts1534_5.2
BFpts1534_5.1
BFpts1532_5.12
SYpte70_5.4
SYptt61_20.1
SYpte66_5.5
SYpte70_5.5
SYptt13_5.1
SYpte93_5.1
SYpte93_5.4
KApts1674_1.1
GTptt901_2.8
GTptt902_2.4
SYptt75_60.16
SYptt75_1000.3
SYptt117_20.1
KApts1680_500.1
SYptt5_40.6
SYptt65_5.1
KApts1703_5.5
KApts1698_5.6
TCptt18_10.4
KApts1681_5.2
KApts1703_5.6
KApts1680_100.11
KApts1680_100.6
KApts1698_5.3
KApts1680_100.1
KApts1682_5.4
BFpts1535_5.5
KApts1682_5.2
BFpts1534_5.20
BFpts1532_5.1
KApts1703_5.11
KApts1682_5.9
KApts1698_5.7
KApts1680_100.2
SYptt117_20.9
SYpte66_5.4
SYptt82_5.1
MEptt2520_2.2
SYpte105_5.1
LBggg314_2.11
SAggg3066_200.4
NDggg3203_2.5
NDggg3203_10.14
NDggg3203_2.4
BQggg61_2.7
GTggg118_2.23
SAggg3066_200.21
SAggg3066_200.24
SAggg3066_200.3
SAggg3066_200.2
BQggg60_2.1
LBggg314_2.1
GTggg509_2.9
GTggg8_2.2
DSggg60_2.3
LBggg1222_10.10
BQggg60_2.5
LBggg314_2.2
LBggg177_2.7
100
100
86
64
81
89
69
100
96
100
100
80
82
63
64
76
C1
G1
C3
G3
C2
G2
0.01

Fig. S6. Maximum likelihood phylogeny of *Laverania* apicoplast sequences. 227 SGA-derived caseinolytic protease M (*clpM*) sequences (390 bp) from 19 blood and 138 fecal samples of sanctuary and wild-living apes are shown, including identical sequences from different samples collected at the same field site (identical sequences from the same sample are excluded). Sequences are labeled and color-coded as in supplementary fig. S1. Asterisks indicate sequences newly generated for this study (for GenBank accession numbers see supplementary table S5, Supplementary Material online). The phylogenetic tree was inferred using PhyML. Bootstrap values (out of 1,000) are shown for major nodes only (the scale bar represents 0.01 substitutions per site).

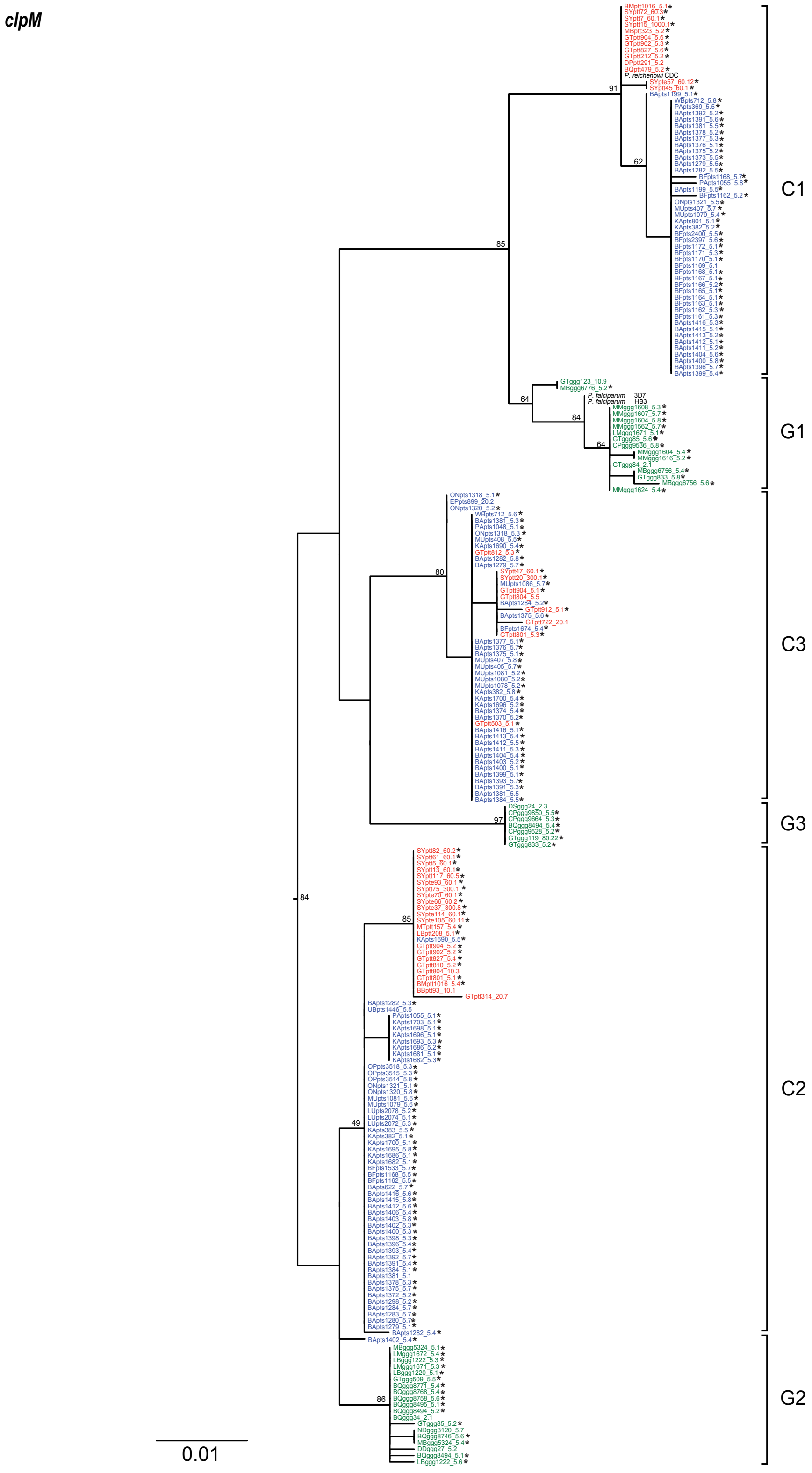
clpM
C1
G1
C3
G3
C2
G2
0.01

Table S1

Chimpanzee and gorilla samples used to derive new *Laverania* sequences

	P. t. ellioti		*P. t. troglodytes*		*P. t. schweinfurthii*		*G. g. gorilla*	
**Sites**[a]	**Samples**	**Individuals**[b]	**Samples**	**Individuals**[b]	**Samples**	**Individuals**[b]	**Samples**	**Individuals**[b]
SY	10	7	20	17				
TC			1	1				
SA							1	1
BM			1	1				
BQ			1	1			15	8
DG			1	1				
GT			14	8			8	4
LB			1	1			4	2
MB			1	1			4	2
ME			1	1				
MT			1	1				
BA					41	11		
BF					19	5		
BI					1	1		
KA					20	5		
LU					5	1		
MU					8	2		
ON					6	2		
OP					5	1		
PA					4	1		
UB					1	1		
WA					1	1		
WB					1	1		
BY							4	2
CP							16	9
DD							10	5
DS							2	1
LM							2	1
MM							13	7
MS							3	2
ND							1	1
NG							1	1
Total	10	7	42	33	112	32	84	46

[a]The location of all sample collection sites is shown in fig. 1, except for SA, which represents a gorilla bushmeat sample of unknown geographic origin.
[b]Except for the SY and TC sanctuaries, numbers of sampled chimpanzees and gorillas were estimated from numbers of fecal samples, taking into consideration host mitochondrial haplotypes and oversampling, as previously reported (Liu et al. 2010).

Table S2

Laverania species composition of ape blood and fecal samples

No	Specimen Code[a]	Sample	*cytB*[b]		*eba165*[b]		*eba175*[b]		*p47*[b]		*ldh*[b]		*clpM*[b]	
			No. (hap)[c]	*Species*[d]	No. (hap)[c]	*Species*[d]	No. (hap)[c]	*Species*[d]	No. (hap)[c]	*Species*[d]	No. (hap)[c]	*Species*[d]	No. (hap)[c]	*Species*[d]
1	SYpte37	Blood	8 (1)	C2	3 (1)	C2	6 (1)	C2	5 (2)	C2	27 (1)	C2	3 (1)	C2
2	SYpte57	Blood	92 (1)	C1	6 (1)	C1	3 (1)	C1	7 (1)	C1	32 (1)	C1	1 (1)	C1
3	SYpte66	Blood	14 (1)	C2	4 (1), 1 (1)	C2			5 (2)	C2	29 (2)	C2	4 (1)	C2
4	SYpte70	RBC[e]	6 (3)	C1, C2	6 (2)	C2	4 (2)	C2	6 (3)	C2	22 (2)	C2	7 (1)	C2
5	SYpte84	Blood	12 (1)	C2	3 (2)	C2	1 (1)	C2	2 (1)	C2				
6	SYpte93	RBC[e]	11 (1)	C2	5 (1)	C2	4 (2)	C2	4 (2)	C2	16 (2)	C2	4 (1)	C2
7	SYpte105	RBC[e]	12 (1)	C2	2 (1)	C2	1 (1)	C2	2 (1)	C2	5 (1)	C2	1 (1)	C2
8	SYpte106	RBC[e]	5 (1)	C2	1 (1)	C2								
9	SYpte107	Blood	2 (1)	C2										
10	SYpte114	Blood	3 (1)	C2					11 (1)	C2	8 (1)	C2	12 (1)	C2
11	SYptt5	Blood	5 (1)	C2	8 (1)	C2	5 (1)	C2	10 (2)	C2	37 (2)	C2	11 (1)	C2
12	SYptt7	Blood	5 (1)	C1	4 (1)	C1	5 (1)	C1	1 (1)	C1	23 (2)	C1	11 (1)	C1
13	SYptt13	Blood	1 (1)	C2	8 (1)	C2	2 (1)	C2	11 (1)	C2	10 (1)	C2	12 (1)	C2
14	SYptt15	Blood	11 (1)	C1					7 (1)	C1			12 (1)	C1
15	SYptt20	Blood	4 (1)	C3	42 (2)	C3	6 (1)	C3	7 (1)	C3	23 (1)	C3	11 (1)	C3
16	SYptt45	Blood	12 (1)	C1	1 (1)	C1			8 (1)	C1	37 (1)	C1	12 (1)	C1
17	SYptt47	Blood	7 (1)	C3									5 (1)	C3
18	SYptt48	Blood							7 (1)	C1				
19	SYptt61	Blood	24 (1)	C2	4 (1)	C2	8 (1)	C2	8 (1)	C2	13 (1)	C2	12 (1)	C2
20	SYptt62	Blood									28 (2)	C2		
21	SYptt63	RBC[e]	11 (1)	C2			6 (2)	C2			13 (1)	C2		
22	SYptt64	Blood	8 (1)	C2					6 (1)	C1				
23	SYptt65	Blood	14 (1)	C2					5 (1)	C2				
24	SYptt72	RBC[e]	6 (2)	C1	8 (1)	C1	6 (2)	C1, C2	8 (1)	C1	10 (2)	C1	7 (1)	C1
25	SYptt75	RBC[e]	15 (3)	C2	5 (1)	C2	3 (2)	C2	53 (4)	C2	29 (4)	C2	9 (1)	C2
26	SYptt79	Blood	1 (1)	C3	5 (1), 1 (1)	C3	4 (1)	C3	1 (1)	C3	2 (2)	C3		
27	SYptt82	RBC[e]	12 (1)	C2	8 (1)	C2	8 (1)	C2	7 (1)	C2	20 (1)	C2	2 (1)	C2
28	SYptt109	Blood	3 (1)	C2										
29	SYptt117	Blood	4 (1)	C2	7 (2)	C2			8 (4)	C2	6 (2)	C2	6 (1)	C2
30	SYptt126	Blood	4 (1)	C3	2 (1)	C3			1 (1)	C3				
31	TCptt18[f]	Blood	101 (2)	C1	2 (2)	C1	4 (1)	C1	11 (4)	C1, C2	28 (1)	C1		
32	SAggg3066[f]	Blood	15 (4)	G1, G2	12 (4)	G2			13 (5)	G2	30 (4)	G2	9 (1)	G2
33	BMptt1016	Faeces	2 (2)	C1, C2	1 (1)	C3	1 (1)	C1	1 (1)	C2	1 (1)	C2	3 (2)	C1, C2
34	BQptt479	Faeces	5 (2)	C1, C2					1 (1)	C1	1 (1)	C2	1 (1)	C1
35	DGptt540	Faeces	20 (8)	C1, C3	4 (1), 2 (2)	C1	11(3), 2(2)	C1, C3	13 (4)	C1	2 (2)	C1	7 (5)	C2, C3
36	GTptt212	Faeces	12 (1)	C1	2 (1), 3 (2)	C1	2 (1)	C1			2 (2)	C1	7 (1)	C1
37	GTptt503	Faeces	3 (2)	C3	1 (1)	C3	1 (1)	C3			1 (1)	C3	8 (1)	C3
38	GTptt706	Faeces	17 (4)	C2, C3							1 (1)	C2		
39	GTptt715	Faeces	2 (2)	C2, C3	1 (1)	C2								
40	GTptt801	Faeces	6 (2)	C2, C3									8 (2)	C2, C3
41	GTptt810	Faeces	3 (2)	C2									4 (1)	C2
42	GTptt812	Faeces	2 (2)	C3									2 (1)	C3
43	GTptt816	Faeces	1 (1)	C2										
44	GTptt824	Faeces	1 (1)	C1										
45	GTptt827	Faeces	3 (2)	C1, C3									3 (2)	C1, C2
46	GTptt901	Faeces	2 (2)	C2	1 (1), 1 (1)	C1, C2			2 (2)	C2				
47	GTptt902	Faeces	5 (3)	C1, C3	2 (2)	C1	2 (2)	C1	3 (3)	C1, C2			4 (2)	C1, C2
48	GTptt904	Faeces	3 (1)	C2									5 (3)	C1-3

49	GTptt912	Faeces	6 (2)	C1, C3			1 (1)	C3	2 (1)	C3	1 (1)	C3	2 (1)	C3
50	LBptt208	Faeces	4 (2)	C1, C2	1 (1)	C2	1 (1)	C2	2 (2)	C2	1 (1)	C2	4 (1)	C2
51	MBptt323	Faeces	5 (2)	C1	1 (1)	C1							6 (1)	C1
52	MEptt2520	Faeces	8 (5)	C1-3	1 (1)	C2			1 (1)	C2				
53	MTptt157	Faeces	4 (3)	C2	1 (1)	C2	6 (4)	C2, C3			5 (3)	C2	5 (1)	C2
54	BApts1199	Faeces	2 (2)	C1, C3									2 (2)	C1
55	BApts1279	Faeces	3 (3)	C1-3					1 (1)	C3			4 (3)	C1-3
56	BApts1280	Faeces	1 (1)	C1					1 (1)	C2			2 (1)	C2
57	BApts1282	Faeces	2 (1)	C2			1 (1)	C1					4 (4)	C1-3
58	BApts1283	Faeces	1 (1)	C3									2 (1)	C2
59	BApts1284	Faeces	6 (3)	C2, C3	1 (1)	C3							2 (2)	C2, C3
60	BApts1286	Faeces							1 (1)	C3				
61	BApts1287	Faeces	2 (2)	C1, C3										
62	BApts1293	Faeces	3 (1)	C1										
63	BApts1294	Faeces	1 (1)	C2										
64	BApts1295	Faeces	2 (2)	C1, C2	1 (1)	C2								
65	BApts1298	Faeces	2 (2)	C2									4 (1)	C2
66	BApts1370	Faeces	1 (1), 1 (1)	C1, C3									1 (1)	C3
67	BApts1372	Faeces	2 (1)	C2									1 (1)	C2
68	BApts1373	Faeces	2 (2), 1 (1)	C1-3									1 (1)	C1
69	BApts1374	Faeces	1 (1)	C3									1 (1)	C3
70	BApts1375	Faeces	9 (4), 3 (0)	C1-3									5 (4)	C1-3
71	BApts1376	Faeces	2 (2)	C2									2 (2)	C1, C3
72	BApts1377	Faeces	1 (1)	C3									4 (2)	C1, C3
73	BApts1378	Faeces	1 (1)	C2									4 (2)	C1, C2
74	BApts1379	Faeces	1 (1)	C1										
75	BApts1381	Faeces	1 (1)	C2									2 (2), 7 (1)	C1-3
76	BApts1384	Faeces	2 (1)	C2									2 (2)	C2, C3
77	BApts1391	Faeces	2 (1)	C2									5 (3)	C1-3
78	BApts1392	Faeces	1 (1)	C3									4 (2)	C1, C2
79	BApts1393	Faeces	1 (1)	C2									2 (2)	C2, C3
80	BApts1396	Faeces	1 (1)	C1									3 (2)	C1, C2
81	BApts1398	Faeces	1 (1)	C3									1 (1)	C2
82	BApts1399	Faeces	2 (2)	C2, C3									4 (2)	C1, C3
83	BApts1400	Faeces	1 (1)	C3									4 (3)	C1-3
84	BApts1402	Faeces	1 (1)	C1	1 (1)	C2							2 (2)	C2
85	BApts1403	Faeces	2 (2)	C1, C3									2 (2)	C2, C3
86	BApts1404	Faeces	2 (1)	C2									3 (2)	C1, C3
87	BApts1406	Faeces	1 (1)	C3									2 (1)	C2
88	BApts1411	Faeces	2 (2)	C3									4 (2)	C1, C3
89	BApts1412	Faeces	1 (1)	C3									3 (3)	C1-3
90	BApts1413	Faeces	9 (6), 2 (1)	C1-3									4 (2)	C1, C3
91	BApts1415	Faeces	3 (3)	C2, C3									3 (2)	C1, C2
92	BApts1416	Faeces	1 (1)	C2									5 (3)	C1-3
93	BApts621	Faeces	2 (1)	C2										
94	BApts622	Faeces	2 (1)	C2									1 (1)	C2
95	BFpts1161	Faeces	3 (2)	C1, C2			1 (1)	C1					4 (1)	C1
96	BFpts1162	Faeces	2 (2)	C1	1 (1), 1 (1)	C1, C2							4 (3)	C1, C2
97	BFpts1163	Faeces	2 (2)	C1									7 (1)	C1
98	BFpts1164	Faeces	5 (3)	C1									8 (1)	C1
99	BFpts1165	Faeces	1 (1)	C1									6 (1)	C1
100	BFpts1166	Faeces	3 (1)	C1									7 (1)	C1
101	BFpts1167	Faeces	2 (2)	C1									5 (1)	C1
102	BFpts1168	Faeces	2 (1)	C1	1 (1)	C2	1 (1)	C2					4 (3)	C1, C2
103	BFpts1169	Faeces	2 (1)	C1									12 (2)	C1
104	BFpts1170	Faeces	2 (1)	C1							1 (1)	C1	7 (1)	C1
105	BFpts1171	Faeces	2 (2), 3 (2)	C1, C3									5 (1)	C1
106	BFpts1172	Faeces	3 (3), 4 (0)	C1, C3									5 (1)	C1

107	BFpts1532	Faeces	2 (2)	C2	1 (1)	C2	1 (1)	C2	2 (2)	C2				
108	BFpts1533	Faeces	1 (1)	C2	1 (1)	C2							1 (1)	C2
109	BFpts1534	Faeces	3 (2)	C2	1 (1)	C2			4 (3)	C2	2 (2)	C2		
110	BFpts1535	Faeces	1 (1)	C2	1 (1)	C2			1 (1)	C2				
111	BFpts2160	Faeces	1 (1)	C3										
112	BFpts2397	Faeces	3 (2)	C1									1 (1)	C1
113	BFpts2400	Faeces	1 (1)	C1	1 (1)	C1							1 (1)	C1
114	BIpts2416	Faeces	1 (1)	C2			1 (1)	C2			1 (1)	C2		
115	KApts1418	Faeces	1 (1)	C1										
116	KApts1663	Faeces	1 (1)	C2										
117	KApts1664	Faeces	1 (1)	C2										
118	KApts1674	Faeces	8(7), 1 (1)	C1-3					1 (1)	C2			1 (1)	C3
119	KApts1679	Faeces	1 (1)	C2										
120	KApts1680	Faeces	32 (8)	C1, C2	2 (1), 15 (4)	C2	8 (6), 2 (1)	C1-3	34 (5)	C2	6 (4)	C2	3 (3)	C2, C3
121	KApts1681	Faeces	19 (1)	C2	4 (2)	C2	3 (2)	C2	2 (1)	C2	1 (1)	C2	8 (1)	C2
122	KApts1682	Faeces	12 (2)	C2	1 (1), 2 (2)	C2	6 (3)	C2	5 (3)	C2	1 (1)	C2	8 (2)	C2
123	KApts1686	Faeces	1 (1)	C2	1 (1)	C2			2 (1)	C3			5 (2)	C2
124	KApts1690	Faeces	8 (6)	C2, C3									2 (2)	C2, C3
125	KApts1693	Faeces	2 (2)	C1, C2									1 (1)	C2
126	KApts1694	Faeces	1 (1)	C2										
127	KApts1695	Faeces	1 (1)	C2									1 (1)	C2
128	KApts1696	Faeces	2 (1)	C2									2 (2)	C2, C3
129	KApts1698	Faeces	7 (3)	C2	4 (2)	C2	4 (2), 1 (1)	C2	5 (4)	C2	5 (5)	C2	7 (1)	C2
130	KApts1700	Faeces	2 (2)	C2, C3									2 (2)	C2, C3
131	KApts1703	Faeces	12 (3)	C2	4 (4)	C2	2 (1)	C2	6 (4)	C2	1 (1)	C2	8 (1)	C2
132	KApts382	Faeces	2 (1), 2 (2)	C1									4 (3)	C1-3
133	KApts383	Faeces	3 (2)	C2									2 (1)	C2
134	KApts801	Faeces	1 (1)	C3									1 (1)	C1
135	LUpts2070	Faeces	1 (1)	C1										
136	LUpts2072	Faeces	1 (1)	C2									3 (1)	C2
137	LUpts2074	Faeces	3 (2), 1 (0)	C2									1 (1)	C2
138	LUpts2078	Faeces	2 (2), 2 (0)	C2									1 (1)	C2
139	LUpts2079	Faeces	1 (1)	C2										
140	MUpts1078	Faeces	1 (1)	C3									2 (1)	C1
141	MUpts1079	Faeces	2 (2)	C2, C3									2 (2)	C1, C2
142	MUpts1080	Faeces	4 (4)	C1-3									1 (1)	C3
143	MUpts1081	Faeces	2 (2)	C1, C2									4 (2)	C2, C3
144	MUpts1086	Faeces	2 (2), 1 (1)	C1, C2									1 (1), 1 (1)	C2, C3
145	MUpts405	Faeces	1 (1)	C3									1 (1)	C3
146	MUpts407	Faeces	1 (1)	C3									2 (2)	C1, C3
147	MUpts408	Faeces	1 (1)	C3									1 (1)	C3
148	ONpts1318	Faeces	1 (1)	C2					1 (1)	C2			2 (2)	C3
149	ONpts1320	Faeces	2 (1)	C3			1 (1)	C3					2 (2)	C2, C3
150	ONpts1321	Faeces	1 (1), 2 (2)	C1, C3			1 (1)	C1	1 (1)	C3			3 (2)	C1, C2
151	ONpts1337	Faeces	1 (1)	C3										
152	ONpts1345	Faeces	1 (1)	C1							1 (1)	C3		
153	ONpts1355	Faeces	2 (2)	C3					1 (1)	C1				
154	OPpts3514	Faeces	1 (1)	C2									1 (1)	C2
155	OPpts3515	Faeces	1 (1)	C2									2 (1)	C2
156	OPpts3518	Faeces	1 (1)	C2									1 (1)	C2
157	OPpts3527	Faeces	1 (1)	C2										
158	OPpts3528	Faeces	1 (1)	C2										
159	PApts1048	Faeces	1 (1)	C3									2 (1)	C3
160	PApts1055	Faeces	2 (2)	C1, C3	1 (1)	C3							2 (2)	C1, C2
161	PApts368	Faeces	3 (1)	C1	1 (1)	C1								
162	PApts369	Faeces	1 (1)	C1									2 (1)	C1
163	UBpts1452	Faeces	1 (1)	C2										
164	WApts519	Faeces	1 (1)	C3										

165	WBpts712	Faeces	2 (2)	C1, C3									2 (2)	C1, C3
166	BQggg60	Faeces	11 (7)	G1-3					2 (2)	G2				
167	BQggg61	Faeces	12 (6)	G1, G2			1 (1)	G2	1 (1)	G2				
168	BQggg8047	Faeces	4 (1)	G2										
169	BQggg8058	Faeces	2 (1)	G1										
170	BQggg8493	Faeces	1 (1)	G3										
171	BQggg8494	Faeces	4 (4)	G1-3									5 (3)	G2, G3
172	BQggg8495	Faeces	5 (5)	G1, G2									3 (1)	G2
173	BQggg8746	Faeces	1 (1)	G2									1 (1)	G2
174	BQggg8750	Faeces	1 (1)	G2										
175	BQggg8752	Faeces	2 (2)	G2			1 (1)	G2						
176	BQggg8758	Faeces	2 (2)	G2									1 (1)	G2
177	BQggg8764	Faeces	6 (5)	G1, G2										
178	BQggg8768	Faeces	6 (3)	G2									2 (1)	G2
179	BQggg8770	Faeces	2 (1)	G2										
180	BQggg8771	Faeces	4 (4)	G1, G2	1 (1)	G2							2 (1)	G2
181	BYggg3814	Faeces	1 (1)	G1										
182	BYggg3821	Faeces	2 (1)	G1										
183	BYggg5016	Faeces	1 (1)	G2										
184	BYggg5029	Faeces	1 (1)	G2										
185	CPggg6665	Faeces	1 (1)	G2										
186	CPggg6673	Faeces	1 (1)	G2										
187	CPggg9521	Faeces	1 (1)	G3										
188	CPggg9524	Faeces	2 (2)	G2, G3										
189	CPggg9528	Faeces	3 (1)	G3	1 (1)	G3							3 (1)	G3
190	CPggg9529	Faeces	5 (2)	G1										
191	CPggg9536	Faeces	2 (1)	G1									1 (1)	G1
192	CPggg9664	Faeces	6 (1)	G3	1 (1)	G3			1 (1)	G3			2 (1)	G3
193	CPggg9668	Faeces	1 (1)	G1										
194	CPggg9669	Faeces	2 (1)	G1										
195	CPggg9671	Faeces	3 (1)	G1										
196	CPggg9678	Faeces	1 (1)	G1										
197	CPggg9681	Faeces	3 (1)	G3										
198	CPggg9682	Faeces	2 (1)	G1										
199	CPggg9687	Faeces	2 (1)	G1										
200	CPggg9850	Faeces	2 (1)	G3									2 (1)	G3
201	DDggg27	Faeces	6 (3)	G1-3					2 (1)	G3			3 (3)	G2, G3
202	DDggg3567	Faeces	9 (1)	G3			1 (1)	G3						
203	DDggg3568	Faeces	8 (1)	G3										
204	DDggg3603	Faeces	3 (1)	G3										
205	DDggg3606	Faeces	1 (1)	G1										
206	DDggg3608	Faeces	3 (2)	G3										
207	DDggg4327	Faeces	1 (1)	G2										
208	DDggg4331	Faeces	11 (1)	G3			1 (1)	G3						
209	DDggg4334	Faeces	1 (1)	G2										
210	DDggg4346	Faeces	2 (1)	G2										
211	DSggg24	Faeces	10 (2)	G3	2 (2)	G2, G3	1 (1)	G3			2 (1)	G3	2 (1)	G3
212	DSggg60	Faeces							1 (1)	G2				
213	GTggg118	Faeces	42 (6)	G1	1 (1)	G1	5 (2), 1 (1)	G1, G2	3 (2)	G1, G2	2 (2)	G1	1 (1)	G1
214	GTggg119	Faeces	31 (3)	G3	1 (1)	G3	3 (1)	G3	3 (1)	G3	2 (1)	G3	22 (1)	G3
215	GTggg509	Faeces	10 (4)	G1, G2	2 (2)	G2			2 (1)	G2	1 (1)	G2	1 (1)	G2
216	GTggg8	Faeces	7 (4)	G2	1 (1)	G2			3 (2)	G2, G3				
217	GTggg10	Faeces	15 (5)	G1	1 (1)	G2	3 (2), 1 (1)	G1, G3			2 (2)	G1	4 (1)	G1
218	GTggg833	Faeces	4 (1)	G3	1 (1)	G3							2 (2)	G1, G3
219	GTggg84	Faeces	8 (3)	G1	1 (1)	G1	3 (2)	G1			1 (1)	G1	7 (1)	G1
220	GTggg85	Faeces									1 (1)	G2	3 (2)	G1, G2
221	LBggg1220	Faeces	12 (9)	G1, G2			2 (1)	G2					1 (1)	G2
222	LBggg1222	Faeces	1 (1)	G2	1 (1)	G2	2 (1), 1 (1)	G2	2 (1)	G2	4 (4)	G2	4 (2)	G2

223	LBggg177	Faeces	4 (3)	G1, G2					1 (1)	G2				
224	LBggg314	Faeces	11 (7)	G1-3					3 (3)	G2				
225	LMggg1671	Faeces	12 (2)	G2							1 (1)	G2	4 (2)	G1, G2
226	LMggg1672	Faeces	6 (3)	G1, G2	1 (1)	G2							2 (1)	G2
227	MBggg5324	Faeces	5 (3)	G1, G2									2 (2)	G2
228	MBggg6746	Faeces	4 (2)	G1, G2										
229	MBggg6756	Faeces	9 (1)	G1									3 (2)	G1
230	MBggg6776	Faeces	3 (1)	G1									1 (1)	G1
231	MMggg1511	Faeces	1 (1)	G3										
232	MMggg1562	Faeces	5 (3)	G1, G2									1 (1)	G1
233	MMggg1598	Faeces	1 (1)	G2										
234	MMggg1601	Faeces	3 (3)	G2										
235	MMggg1603	Faeces	1 (1)	G1										
236	MMggg1604	Faeces	5 (3)	G1, G2									2 (2)	G1
237	MMggg1607	Faeces	1 (1)	G2									1 (1)	G1
238	MMggg1608	Faeces	1 (1)	G1									1 (1)	G1
239	MMggg1609	Faeces	1 (1)	G2										
240	MMggg1614	Faeces	2 (2)	G2										
241	MMggg1616	Faeces	1 (1)	G1									3 (1)	G1
242	MMggg1622	Faeces	3 (1)	G1										
243	MMggg1624	Faeces	1 (1)	G1									1 (1)	G1
244	MSggg7183	Faeces	4 (2)	G1, G3										
245	MSggg7193	Faeces	2 (1)	G2										
246	MSggg7212	Faeces	2 (2)	G2										
247	NDggg3203	Faeces	12 (4)	G1-3	1 (1)	G1	1 (1)	G1	6 (6)	G1-3			1 (1)	G1
248	NGggg4475	Faeces	1 (1)	G2										

[a]Blood samples were collected opportunistically from individually known chimpanzees housed at the Sanaga Yong Chimpanzee Rescue Center (SY) and the Tchimpounga Chimpanzee Rehabilitation Center (TC). One gorilla blood sample was obtained from a bushmeat carcass of unknown geographic origin (SA) confiscated by the anti-poaching program of the Cameroonian Ministry of Environment and Forestry. Fecal samples were collected from wild-living apes, with their species and subspecies origin determined by host mitochondrial DNA analysis (*ptt, P. t. troglodytes; pte, P. t. ellioti; pts, P. t. schweinfurthii; ggg, G. g. gorilla*); a two-letter code indicates the field site of origin (fig. 1). Samples highlighted in red were newly characterized in this study and subjected to single template amplification of *Laverania* parasite sequences.

[b]Single template amplified loci of *Laverania* mitochondrial (*cytB*), nuclear (*eba165, eba175, p47, and ldh*) and apicoplast (*clpM*) genes. Sequences newly derived in this study are highlighted in red.

[c]No., number of single genome amplification (SGA)-derived sequences, with brackets indicating the number of distinguishable haplotypes (hap). Sequences indicated in black have previously been published (Liu et al. 2010; Wanaguru et al. 2013; Sundararaman et al. 2016). Sequences in red were newly derived for this study. See supplementary table S5 for GenBank accession numbers.

[d]Ape *Laverania* species present in sample: C1, *P. reichenowi*; C2, *P. gaboni*; C3, *P. billcollinsi*; G1, *P. praefalciparum*; G2, *P. alderi*; G3, *P. blacklocki.*

[e]RBC, red blood cells were purified by Lymphoprep density gradient centrifugation.

[f]DNA from this blood sample was pre-amplified by select whole genome amplification (SWGA) prior to SGA analysis as described (Sundararaman et al. 2016).

Table S3

Single template PCR-derived ape *Laverania* sequences from fecal and blood samples

	cytB[a]		***eba165***[a]		***eba175***[a]		***p47***[a]		***ldh***[a]		***clpM***[a]	
**Sample**	**New (hap)**[b]	**Pub (hap)**[c]	**New (hap)**[b]	**Pub (hap)**[c]	**New (hap)**[b]	**Pub (hap)**[c]	**New (hap)**[b]	**Pub (hap)**[c]	**New (hap)**[b]	**Pub (hap)**[c]	**New (hap)**[b]	**Pub (hap)**[c]
Feces	413 (253)	811 (425)	52 (36)	38 (33)	31 (25)	63 (38)	123 (69)	n/a	12 (10)	46 (40)	393 (190)	123 (69)
Blood	38 (6)	415 (29)	114 (24)	34 (8)	76 (22)	n/a	214 (45)	n/a	448 (38)	0	151 (20)	0
Total	1,677 (713)		238 (101)		170 (85)		337 (114)		506 (88)		667 (279)	

[a]Single template amplified loci of *Laverania* mitochondrial (*cytB*), nuclear (*eba165, eba175, p47, ldh*), and apicoplast (*clpM*) genes (the *clpM* gene, which encodes the Clp chaperone PfC10_API0060, has previously been called *clpC* (Liu et al. 2010).

[b]Number of new SGA-derived *Laverania* sequences, with brackets indicating the number of distinguishable haplotypes.

[c]Number of previously reported SGA-derived *Laverania* sequences, with brackets indicating the number of distinguishable haplotypes (Liu et al. 2010; Wanaguru et al. 2013; Sundararaman et al. 2016).

Table S4

Genealogical sorting index (gsi) values for C2 samples from west and east central Africa

Gene	West	East
cytB	0.25**	0.93**
clpM	0.79**	0.00
eba165	0.44*	0.51**
eba175	0.00	0.25
ldh	0.00	0.02
p47	0.44**	0.14
organelle	0.52	0.46
nuclear	0.22	0.23

The analyses refer to C2 sequences shown in figs. 2, 3 and 4. Samples from *P. t. troglodytes* and *P. t. ellioti* were together classified as from west central Africa (West); samples from *P. t. schweinfurthii* were classified as from east central Africa (East) (fig. 1). Values of the genealogical sorting index (gsi; Cummings et al. 2008) range from 0 (where the samples show no segregation) to 1 (with complete monophyly); values significantly higher than expected from random segregation are noted (* $p < 0.05$; ** $p < 0.01$). Average values for organelle (*cytB*, *clpM*) and nuclear (*eba165*, *eba175*, *ldh*, *p47*) gene sequences are given on the bottom.

Values for both organelle gene sequences show significant evidence of segregation, whereas values for two of the four nuclear genes show no significant evidence of segregation. The average gsi values for organelle genes are at least 2 times higher than those for nuclear genes.

The gsi values were calculated from the maximum likelihood phylogenies for the C2 sequences, with zero branch lengths collapsed to polytomies, using the genealogicalSorting R package (http://molecularevolution.org/software/phylogenetics/gsi). Statistical significance was assessed by

randomly permuting character states (West or East) across the tips of the tree 10,000 times; p values were adjusted for the fact that 12 tests were performed.

Note that, for *cytB*, a single sequence from the West group lies within the subclade of sequences from the East group (fig. 2). If this sequence lay outside the East group (which could occur with one nucleotide difference), the gsi value for the West group would be 0.93 (and that for the East group would be 1.0). Similarly, for *clpM*, the gsi value for the East group would be much higher (0.79), but for a single sequence from the East group lying within the subclade of sequences from the West group. There are no individual sequences that have similar influence on the gsi values for nuclear genes.

As in clade C2, organelle gene sequences from clade C1 exhibit signs of segregation into East and West subclades (figs. 2 and 4), while nuclear gene sequences clearly do not (fig. 3); however, we had too few East sequences for the nuclear genes to generate comparable gsi values for clade C1.

Table S5

GenBank accession numbers for SGA-derived *Laverania* sequences

Sample	*cytB*[a]	Accession No.[b]	*eba165*[a]	Accession No.[b]	*eba175*[a]	Accession No.[b]	*p47*[a]	Accession No.[b]	*ldh*[a]	Accession No.[b]	*clpM*[a]	Accession No.[b]
**SYpte37**					SYptt37_200.7	=KF568935	SYpte37_10.1	KU665787	SYpte37_40.3	=HM235133	SYpte37_300.8	=HM235145
							SYpte37_10.7	KU665788				
**SYpte57**			SYpte57_20.2	=SYptt45_500.7	SYpte57_40.1	=KF568910	SYpte57_10.1	KU665789	SYpte57_20.2	=HM235126	SYpte57_60.12	KU665661
**SYpte66**			SYpte66_20.1	KT824361			SYpte66_5.4	=MEptt2520_2.2	SYpte66_20.1	=SYptt82_20.1	SYpte66_60.2	=HM235145
							SYpte66_5.5	=SYptt13_5.1	SYpte66_20.4	=HM235130		
**SYpte70**			SYpte70_5.1	=SYptt5_20.1	SYpte70_10.3	KU665715	SYpte70_5.1	KU665790	SYpte70_20.2	=SYptt82_20.1	SYpte70_60.1	=HM235145
			SYpte70_5.4	KT824362	SYpte70_10.4	KU665716	SYpte70_5.4	KU665791	SYpte70_20.4	KU665725		
							SYpte70_5.5	=SYptt13_5.1				
**SYpte84**	SYpte84_5.1	=HM235353	SYpte84_5.1	=KT824365	SYpte84_10.8	=KF568913	SYpte84_5.4	=BMptt1016_5.10				
			SYpte84_5.11	=SYptt5_20.1								
**SYpte93**			SYpte93_5.2	=KT824365	SYpte93_10.3	KU665717	SYpte93_5.1	=SYptt13_5.1	SYpte93_20.1	=HM235130	SYpte93_60.1	=HM235145
					SYpte93_10.5	=KF568927	SYpte93_5.4	KU665792	SYpte93_20.4	KU665726		
**SYpte105**	SYpte105_5.1	=HM235353	SYpte105_5.2	=SYptt5_20.1	SYpte105_10.3	=KF568935	SYpte105_5.1	=MEptt2520_2.2	SYpte105_20.5	=SYptt82_20.1	SYpte105_60.11	=HM235145
**SYpte106**	SYpte106_5.1	=KT824304	SYpte106_5.3	=KT824365								
**SYpte107**	SYpte107_5.1	=KT824304										
**SYpte114**							SYpte114_20.1	=SYpte70_5.1	SYpte114_20.4	=SYpte70_20.4	SYpte114_60.1	=HM235145
**SYptt5**			SYptt5_20.1	KT824369	SYptt5_40.2	=KF568933	SYptt5_5.1	=SYpte70_5.1	SYptt5_20.1	KU665729	SYptt5_60.1	=HM235145
							SYptt5_40.6	KU665798	SYptt5_20.2	KU665730		
**SYptt7**			SYptt7_5.2	KT824372	SYptt7_40.2	=KF568910	SYptt7_5.7	=BQptt479_2.8	SYptt7_20.2	=HM235135	SYptt7_60.1	=HM235153
									SYptt7_20.7	KU665733		
**SYptt13**					SYptt13_40.2	=KF568913	SYptt13_5.1	KU665795	SYptt13_20.1	=HM235130	SYptt13_60.1	=HM235145
**SYptt15**							SYptt15_20.1	KU665796			SYptt15_1000.1	=HM235153
**SYptt20**			SYptt20_100.20	KT824366	SYptt20_40.1	=KF568909	SYptt20_10.1	KU665797	SYptt20_20.2	=HM235129	SYptt20_300.1	=HM235162
			SYptt20_500.1	KT824367								
**SYptt45**			SYptt45_500.7	KT824368			SYptt45_10.1	=SYpte57_10.1	SYptt45_20.1	KU665728	SYptt45_60.1	=SYpte57_60.12
**SYptt47**											SYptt47_60.1	=HM235162
**SYptt48**							SYptt48_5.1	=SYptt15_20.1				
**SYptt61**					SYptt61_40.1	KU665718	SYptt61_20.1	=SYptt13_5.1	SYptt61_20.4	=HM235130	SYptt61_60.1	=HM235145
**SYptt62**									SYptt62_10.7	=HM235130		
									SYptt62_10.12	=HM235139		
**SYptt63**					SYptt63_10.2	=KF568927			SYptt63_20.4	=HM235130		
					SYptt63_10.8	=SYpte93_10.3						
**SYptt64**							SYptt64_5.1	=BQptt479_2.8				
**SYptt65**							SYptt65_5.1	=SYptt5_40.6				
**SYptt72**					SYptt72_40.2	=KF568935	SYptt72_5.1	KU665799	SYptt72_20.1	KU665731	SYptt72_60.3	=HM235153
					SYptt72_40.4	=GTptt902_10.11			SYptt72_20.11	KU665732		
**SYptt75**			SYptt75_20.2	KT824370	SYptt75_40.5	=KF568913	SYptt75_60.16	KU665801	SYptt75_100.3	=HM235130	SYptt75_300.1	=HM235145
							SYptt75_500.17	=BMptt1016_5.10	SYptt75_100.6	=SYptt82_20.1		
							SYptt75_1000.1	=SYpte70_5.1	SYptt75_100.13	=HM235139		
					SYptt75_40.7	KU665719	SYptt75_1000.3	KU665800	SYptt75_100.20	=SYptt117_20.1		
**SYptt79**			SYptt79_5.8	= SYptt20_100.20	SYptt79_10.2	=KF568909	SYptt79_5.5	=SYptt20_10.1	SYptt79_20.4	=HM235141		
									SYptt79_20.7	=HM235129		
**SYptt82**			SYptt82_5.1	=KT824360	SYptt82_40.1	=KF568935	SYptt82_5.1	=MEptt2520_2.2	SYptt82_20.1	KU665734	SYptt82_60.2	=HM235145

**SYptt109**	SYptt109_5.1	=HM235353										
**SYptt117**			SYptt117_10.1	=KT824365			SYptt117_10.8	KU665793	SYptt117_20.1	KU665727	SYptt117_60.5	=HM235145
							SYptt117_20.1	=SYptt75_1000.3	SYptt117_20.4	=HM235130		
							SYptt117_20.4	=SYpte70_5.1				
			SYptt117_10.3	KT824363			SYptt117_20.9	KU665794				
**SYptt126**	SYptt126_5.1	KT824305	SYptt126_10.12	KT824364			SYptt126_20.10	=SYptt20_10.1				
**TCptt18**					TCptt18_60.1	=KF568910	TCptt18_10.4	=KApts1703_5.5	TCptt18_20.1	KU665735		
							TCptt18_60.1	KU665802				
							TCptt18_60.3	KU665803				
							TCptt18_60.6	=SYpte57_10.1				
**SAggg3066**			SAggg3066_200.1	KU665702			SAggg3066_200.2	KU665783	SAggg3066_30.1	=HM235124	SAggg3066_100.6A	=LBggg1222_5.6
			SAggg3066_200.8	KU665704			SAggg3066_200.3	KU665785	SAggg3066_30.3	=GTggg509_5.11		
			SAggg3066_200.9	KU665705			SAggg3066_200.4	KU665786	SAggg3066_30.5	=LMggg1671_5.9		
			SAggg3066_200.24	KU665703			SAggg3066_200.21	=NDggg3203_2.5	SAggg3066_200.1	KU665724		
							SAggg3066_200.24	KU665784				
**BMptt1016**							BMptt1016_5.10	KU665744	BMptt1016_5.7	KU665720	BMptt1016_5.1	=HM235153
											BMptt1016_5.4	=HM235145
**BQptt479**	BQptt479_5.2	=HM234980					BQptt479_2.8	KU665747			BQptt479_5.2	=HM235153
	BQptt479_5.3	=HM235353										
**DGptt540**			DGptt540_40.4	=KT824352	DGptt540_2.1	KU665709	DGptt540_10.1	=GTptt902_2.8				
			DGptt540_40.7	=SYptt7_5.2	DGptt540_2.4	=KF568925	DGptt540_10.2	=BQptt479_2.8				
							DGptt540_10.3	=ONpts1355_5.5				
							DGptt540_10.6	KU665749				
**GTptt212**			GTptt212_5.1	=KT824352							GTptt212_5.2	=HM235153
			GTptt212_5.7	KT824336								
**GTptt503**											GTptt503_5.1	=HM235144
**GTptt706**									GTptt706_5.11	=HM235139		
**GTptt715**			GTptt715_5.8	KT824339								
**GTptt801**	GTptt801_60.3	=HM234993									GTptt801_5.1	=HM235145
	GTptt801_60.4	KU665678									GTptt801_5.3	=HM235162
**GTptt810**	GTptt810_60.3	=HM235109									GTptt810_5.2	=HM235145
	GTptt810_60.5	=HM235353										
**GTptt812**	GTptt812_5.4	=HM235018									GTptt812_5.3	=HM235144
	GTptt812_5.8	=HM234993										
**GTptt816**	GTptt816_30.10	KU665679										
**GTptt824**	GTptt824_5.1	=HM234980										
**GTptt827**	GTptt827_30.9	=HM234980									GTptt827_5.4	=HM235145
	GTptt827_30.11	=HM234993									GTptt827_5.6	=HM235153
**GTptt901**			GTptt901_5.5	KT824340			GTptt901_2.3	KU665756				
							GTptt901_2.8	KU665755				
**GTptt902**					GTptt902_10.1	=KF568910	GTptt902_2.1	=BQptt479_2.8			GTptt902_5.2	=HM235145
					GTptt902_10.11	KU665711	GTptt902_2.4	=GTptt901_2.8			GTptt902_5.3	=HM235153
							GTptt902_2.8	KU665757				
**GTptt904**	GTptt904_30.5	=HM235353									GTptt904_5.1	=HM235162
											GTptt904_5.2	=HM235145
											GTptt904_5.6	=HM235153
**GTptt912**	GTptt912_30.2	=HM234993			GTptt912_10.3	=KF568909	GTptt912_2.3	KU665758	GTptt912_5.12	=HM235129	GTptt912_5.1	KU665654
	GTptt912_30.8	=HM234980										
**LBptt208**							LBptt208_5.2	KU665772	LBptt208_1.1	KU665722	LBptt208_5.1	=HM235145

							LBptt208_5.7	=SYpte70_5.1		
**MBptt323**									MBptt323_5.2	=HM235153
**MEptt2520**			MEptt2520_5.1	KT824353			MEptt2520_2.2	KU665773		
**MTptt157**			MTptt157_5.5	KT824354					MTptt157_5.4	=HM235145
**BApts1199**	BApts1199_5.4	KU665662							BApts1199_5.1	KU665647
	BApts1199_60.9	=HM235393							BApts1199_5.5	=HM235146
**BApts1279**	BApts1279_5.4	=HM235391					BApts1279_5.9	KU665736	BApts1279_5.1	=HM235168
	BApts1279_5.5	=HM235390							BApts1279_5.5	=HM235146
	BApts1279_5.6	KU665663							BApts1279_5.7	=HM235144
**BApts1280**	BApts1280_5.1	=HM235397					BApts1280_5.11	KU665737	BApts1280_5.7	=HM235168
**BApts1282**	BApts1282_5.4	=BFpts1533_5.3			BApts1282_2.4	KU665706			BApts1282_5.3	=HM235168
									BApts1282_5.4	KU665648
									BApts1282_5.5	=HM235146
									BApts1282_5.8	=HM235144
**BApts1283**	BApts1283_5.3	KU665664							BApts1283_5.7	=HM235168
**BApts1284**	BApts1284_5.2	=HM235108	BApts1284_5.3	KT824313					BApts1284_5.2	=HM235162
	BApts1284_5.3	=KApts383_5.3							BApts1284_5.7	=HM235168
	BApts1284_5.10	=BFpts1533_5.3								
**BApts1286**							BApts1286_5.4	KU665738		
**BApts1287**	BApts1287_5.2	=HM235397								
	BApts1287_5.4	=HM235108								
**BApts1293**	BApts1293_6.12	=HM235397								
**BApts1294**	BApts1294_5.3	=BApts1295_5.4								
**BApts1295**	BApts1295_5.1	=HM235397	BApts1295_5.1	KT824314						
	BApts1295_5.4	KT824281								
**BApts1298**	BApts1298_5.1	KU665665							BApts1298_5.2	=HM235168
	BApts1298_5.2	KU665666								
**BApts1370**	BApts1370_5.3	=HM235108							BApts1370_5.2	=HM235144
**BApts1372**	BApts1372_5.2	=BFpts1533_5.3							BApts1372_5.2	=HM235168
**BApts1373**	BApts1373_5.3	=HM235108							BApts1373_5.5	=HM235146
**BApts1374**	BApts1374_5.3	=HM235108							BApts1374_5.4	=HM235144
**BApts1375**									BApts1375_5.1	=HM235144
									BApts1375_5.2	=HM235146
									BApts1375_5.6	=HM235162
									BApts1375_5.7	=HM235168
**BApts1376**	BApts1376_5.3	=KApts383_5.3							BApts1376_5.1	=HM235146
	BApts1376_5.4	=HM235050							BApts1376_5.7	=HM235144
**BApts1377**	BApts1377_60.7	KU665667							BApts1377_5.1	=HM235144
									BApts1377_5.3	=HM235146
**BApts1378**	BApts1378_5.4	=BFpts1533_5.3							BApts1378_5.2	=HM235146
									BApts1378_5.3	=HM235168
**BApts1379**	BApts1379_5.3	=HM235397								
**BApts1381**	BApts1381_5.4	=BFpts1533_5.3							BApts1381_5.5	=HM235146
**BApts1384**	BApts1384_5.1	=BFpts1533_5.3							BApts1384_5.1	=HM235168
									BApts1384_5.5	=HM235144
**BApts1391**	BApts1391_60.2	=BFpts1533_5.3							BApts1391_5.3	=HM235144
									BApts1391_5.4	=HM235168
									BApts1391_5.6	=HM235146
**BApts1392**	BApts1392_5.3	=HM235108							BApts1392_5.2	=HM235146

							BApts1392_5.7	=HM235168
**BApts1393**	BApts1393_5.4	=BFpts1533_5.3					BApts1393_5.4	=HM235168
							BApts1393_5.7	=HM235144
**BApts1396**	BApts1396_5.4	=HM235397					BApts1396_5.4	=HM235168
							BApts1396_5.7	=HM235146
**BApts1398**	BApts1398_5.4	=HM235108					BApts1398_5.3	=HM235168
**BApts1399**	BApts1399_5.2	=BFpts1533_5.3					BApts1399_5.1	=HM235144
	BApts1399_5.3	=HM235108					BApts1399_5.4	=HM235146
**BApts1400**	BApts1400_60.8	=BApts1377_60.7					BApts1400_5.1	=HM235144
							BApts1400_5.3	=HM235168
							BApts1400_5.8	=HM235146
**BApts1402**			BApts1402_5.8	KT824315			BApts1402_5.3	=HM235168
							BApts1402_5.4	KU665649
**BApts1403**	BApts1403_5.3	=HM235397					BApts1403_5.2	=HM235144
	BApts1403_5.4	=HM235108					BApts1403_5.8	=HM235168
**BApts1404**	BApts1404_5.1	=BFpts1533_5.3					BApts1404_5.4	=HM235144
							BApts1404_5.6	=HM235146
**BApts1406**	BApts1406_5.2	KU665668					BApts1406_5.4	=HM235168
**BApts1411**	BApts1411_5.1	=BApts1406_5.2					BApts1411_5.2	=HM235146
	BApts1411_5.2	=HM235108					BApts1411_5.3	=HM235144
**BApts1412**	BApts1412_5.2	=HM235108					BApts1412_5.1	=HM235146
							BApts1412_5.5	=HM235144
							BApts1412_5.6	=HM235168
**BApts1413**	BApts1413_5.3	=HM235108					BApts1413_5.2	=HM235146
							BApts1413_5.4	=HM235144
**BApts1415**	BApts1415_5.1	=HM235108					BApts1415_5.1	=HM235146
	BApts1415_5.2	=BApts1377_60.7					BApts1415_5.8	=HM235168
	BApts1415_5.3	=BFpts1533_5.3						
**BApts1416**	BApts1416_5.4	=BFpts1533_5.3					BApts1416_5.1	=HM235144
							BApts1416_5.3	=HM235146
							BApts1416_5.6	=HM235168
**BApts621**	BApts621_5.3	=KApts383_5.3						
**BApts622**	BApts622_5.3	=KApts383_5.3					BApts622_5.7	=HM235168
**BFpts1161**	BFpts1161_5.2	=HM235397			BFpts1161_2.4	=BApts1282_2.4	BFpts1161_5.3	=HM235146
	BFpts1161_5.3	=HM235050						
**BFpts1162**			BFpts1162_5.6	KT824317			BFpts1162_5.2	KU665650
							BFpts1162_5.3	=HM235146
							BFpts1162_5.5	=HM235168
**BFpts1163**	BFpts1163_5.2	KU665669					BFpts1163_5.1	=HM235146
	BFpts1163_5.3	=HM235391						
**BFpts1164**	BFpts1164_5.4	=BFpts1163_5.2					BFpts1164_5.1	=HM235146
	BFpts1164_120.7	=HM235397						
	BFpts1164_120.10	=HM235393						
**BFpts1165**	BFpts1165_5.2	=BFpts1163_5.2					BFpts1165_5.1	=HM235146
**BFpts1166**	BFpts1166_5.3	=HM235397					BFpts1166_5.2	=HM235146
**BFpts1167**	BFpts1167_5.2	=HM235397					BFpts1167_5.1	=HM235146
	BFpts1167_5.3	=HM235391						
**BFpts1168**	BFpts1168_5.1	KT824283	BFpts1168_5.4	KT824318	BFpts1168_2.2	KU6657067	BFpts1168_5.1	=HM235146
							BFpts1168_5.5	=HM235168

											BFpts1168_5.7	KU665651
**BFpts1169**	BFpts1169_5.2	=HM235391										
**BFpts1170**	BFpts1170_5.2	=HM235397									BFpts1170_5.1	=HM235146
**BFpts1171**	BFpts1171_5.1	KU665670									BFpts1171_5.3	=HM235146
	BFpts1171_5.2	=HM235397										
**BFpts1172**											BFpts1172_5.1	=HM235146
**BFpts1532**	BFpts1532_5.1	=BFpts1533_5.3	BFpts1532_5.11	=KApts1680_60.7	BFpts1532_10.10	KU665708	BFpts1532_5.1	KU665740				
	BFpts1532_5.2	KT824284					BFpts1532_5.12	KU665739				
**BFpts1533**	BFpts1533_5.3	KT824285	BFpts1533_5.1	=BFpts1168_5.4							BFpts1533_5.7	=HM235168
**BFpts1534**							BFpts1534_5.1	=BFpts1532_5.12				
							BFpts1534_5.2	KU665741				
							BFpts1534_5.20	KU665742				
**BFpts1535**	BFpts1535_5.4	=KApts383_5.3	BFpts1535_5.4	KT824320			BFpts1535_5.5	KU665743				
**BFpts2160**	BFpts2160_5.2	=HM235108										
**BFpts2397**	BFpts2397_5.1	=HM235402									BFpts2397_5.6	=HM235146
	BFpts2397_5.2	=HM235397										
**BFpts2400**	BFpts2400_5.2	=HM235397	BFpts2400_5.7	KT824321							BFpts2400_5.5	=HM235146
**BIpts2416**	BIpts2416_5.1	=BFpts1533_5.3										
**KApts1418**	KApts1418_5.3	=HM235397										
**KApts1663**	KApts1663_5.3	=HM235050										
**KApts1664**	KApts1664_5.4	=BFpts1533_5.3										
**KApts1674**	KApts1674_5.3	KU665680					KApts1674_1.1	KU665759			KApts1674_5.4	=HM235162
**KApts1679**	KApts1679_5.3	=HM235050										
**KApts1680**			KApts1680_60.1	=KApts1703_60.5	KApts1680_5.10	KU665712	KApts1680_100.1	KU665760				
			KApts1680_60.2	=KApts1686_5.2			KApts1680_100.2	KU665761				
			KApts1680_60.7	KT824343			KApts1680_100.6	KU665762				
			KApts1680_120.2	=KT824344			KApts1680_100.11	=KApts1681_5.2				
							KApts1680_500.1	=KApts1703_5.5				
**KApts1681**	KApts1681_120.6	=HM235050	KApts1681_60.5	=KApts1686_5.2	KApts1681_60.1	=KF568935	KApts1681_5.2	KU665763	KApts1681_1.1	=HM235139	KApts1681_5.1	=PApts1055_5.1
			KApts1681_60.7	=KApts1698_10.3	KApts1681_60.4	=KF568911						
**KApts1682**			KApts1682_60.2	=KApts1698_10.3	KApts1682_120.2	KU665713	KApts1682_5.2	=KApts1681_5.2	KApts1682_1.1	=HM235139	KApts1682_5.1	=HM235168
			KApts1682_60.5	=KApts1686_5.2	KApts1682_120.6	=KF568935	KApts1682_5.4	=KApts1680_100.1			KApts1682_5.3	=PApts1055_5.1
					KApts1682_120.10	=KF568913	KApts1682_5.9	KU665764				
**KApts1686**	KApts1686_5.1	=HM235050	KApts1686_5.2	KT824345			KApts1686_5.3	=ONpts1321_5.5			KApts1686_5.1	=HM235168
											KApts1686_5.2	=PApts1055_5.1
**KApts1690**	KApts1690_5.1	=BFpts1533_5.3									KApts1690_5.4	=HM235144
	KApts1690_5.3	=HM235108									KApts1690_5.5	=HM235145
	KApts1690_5.4	=HM234976										
	KApts1690_5.5	=KApts383_5.3										
	KApts1690_5.8	=HM235050										
	KApts1690_5.15	KU665681										
**KApts1693**	KApts1693_5.1	=HM235052									KApts1693_5.3	=PApts1055_5.1
	KApts1693_5.3	=HM235393										
**KApts1694**	KApts1694_5.3	=HM235050										
**KApts1695**	KApts1695_5.3	=HM235050									KApts1695_5.8	=HM235168
**KApts1696**	KApts1696_5.4	=HM235050									KApts1696_5.1	=PApts1055_5.1
											KApts1696_5.2	=HM235144
**KApts1698**	KApts1698_120.2	=HM235050	KApts1698_10.3	KT824346	KApts1698_5.1	=KF568935	KApts1698_5.3	=KApts1680_100.1			KApts1698_5.1	=PApts1055_5.1

	KApts1698_120.3	KT824295	KApts1698_10.9	=KT824344			KApts1698_5.6	=KApts1703_5.5				
	KApts1698_120.8	=KApts383_5.3					KApts1698_5.7	=KApts1680_100.2				
							KApts1698_5.9	KU665765				
**KApts1700**	KApts1700_5.2	=HM235108									KApts1700_5.1	=HM235168
	KApts1700_5.3	=HM235050									KApts1700_5.4	=HM235144
**KApts1703**	KApts1703_120.2	=HM235050	KApts1703_5.1	=KApts1686_5.2	KApts1703_5.3	=KF568935	KApts1703_5.5	KU665766	KApts1703_1.1	=HM235130	KApts1703_5.1	=PApts1055_5.1
	KApts1703_120.5	KT824296	KApts1703_5.4	=KT824344			KApts1703_5.6	=KApts1681_5.2				
	KApts1703_120.7	=KApts383_5.3	KApts1703_60.2	KT824347			KApts1703_5.11	=KApts1680_100.2				
			KApts1703_60.5	KT824348			KApts1703_5.12	KU665767				
**KApts382**	KApts382_5.2	=HM235391									KApts382_5.1	=HM235168
	KApts382_5.4	=HM235397									KApts382_5.2	=HM235146
											KApts382_5.8	=HM235144
**KApts383**	KApts383_5.1	=BFpts1533_5.3									KApts383_5.5	=HM235168
	KApts383_5.3	KU665682										
**KApts801**	KApts801_5.2	KU665683									KApts801_5.1	=HM235146
**LUpts2070**	LUpts2070_5.2	=HM235402										
**LUpts2072**	LUpts2072_5.2	=BFpts1533_5.3									LUpts2072_5.3	=HM235168
**LUpts2074**											LUpts2074_5.1	=HM235168
**LUpts2078**											LUpts2078_5.2	=HM235168
**LUpts2079**	LUpts2079_5.3	KU665684										
**MUpts1078**	MUpts1078_60.8	=HM235108									MUpts1078_5.2	=HM235144
**MUpts1079**	MUpts1079_5.3	=KApts383_5.3									MUpts1079_5.4	=HM235146
	MUpts1079_5.4	=WBpts712_5.2									MUpts1079_5.6	=HM235168
**MUpts1080**	MUpts1080_5.3	KU665691									MUpts1080_5.2	=HM235144
	MUpts1080_5.4	=KApts383_5.3										
	MUpts1080_60.4	=HM235397										
	MUpts1080_60.7	=HM235402										
**MUpts1081**	MUpts1081_5.1	KU665692									MUpts1081_5.2	=HM235144
	MUpts1081_5.4	=HM235397									MUpts1081_5.6	=HM235168
**MUpts1086**	MUpts1086_5.2	=KApts383_5.3									MUpts1086_5.7	=HM235162
**MUpts405**	MUpts405_5.2	=HM235390									MUpts405_5.7	=HM235144
**MUpts407**	MUpts407_5.3	=HM234976									MUpts407_5.7	=HM235146
											MUpts407_5.8	=HM235144
**MUpts408**	MUpts408_5.2	=HM235390									MUpts408_5.5	=HM235144
**ONpts1318**	ONpts1318_5.1	=BFpts1533_5.3					ONpts1318_10.12	KU665780			ONpts1318_5.1	=HM235156
											ONpts1318_5.3	=HM235144
**ONpts1320**	ONpts1320_5.3	=HM234993			ONpts1320_2.2	=KF568909					ONpts1320_5.2	=HM235156
											ONpts1320_5.8	=HM235168
**ONpts1321**	ONpts1321_5.1	KU665693			ONpts1321_10.2	KU665714	ONpts1321_5.5	KU665781			ONpts1321_5.1	=HM235168
	ONpts1321_5.2	=HM235402									ONpts1321_5.5	=HM235146
**ONpts1337**	ONpts1337_5.2	=HM235108										
**ONpts1345**	ONpts1345_5.4	KU665694										
**ONpts1355**	ONpts1355_5.2	=HM234993					ONpts1355_5.5	KU665782				
	ONpts1355_5.3	KU665695										
**OPpts3514**	OPpts3514_5.2	=KApts383_5.3									OPpts3514_5.8	=HM235168
**OPpts3515**	OPpts3515_5.1	=KApts383_5.3									OPpts3515_5.3	=HM235168
**OPpts3518**	OPpts3518_5.2	=BFpts1533_5.3									OPpts3518_5.3	=HM235168
**OPpts3527**	OPpts3527_5.3	=BFpts1533_5.3										
**OPpts3528**	OPpts3528_5.3	KU665696										

**PApts1048**	PApts1048_5.1	=HM234976							PApts1048_5.1	=HM235144
**PApts1055**									PApts1055_5.1	KU665659
									PApts1055_5.8	KU665660
**PApts368**	PApts368_5.6	KU665697	PApts368_5.6	KU665701						
**PApts369**	PApts369_5.4	=HM235391							PApts369_5.5	=HM235146
**UBpts1452**	UBpts1452_5.3	KU665698								
**WApts519**	WApts519_5.4	=HM235108								
**WBpts712**	WBpts712_5.1	KU665699							WBpts712_5.6	=HM235144
	WBpts712_5.2	KU665700							WBpts712_5.8	=HM235146
**BQggg60**							BQggg60_2.1	KU665745		
							BQggg60_2.5	=LBggg314_2.2		
**BQggg61**							BQggg61_2.7	KU665746		
**BQggg8047**	BQggg8047_5.1	=HM234986								
**BQggg8058**	BQggg8058_5.7	=HM235041								
**BQggg8493**	BQggg8493_30.5	=HM235064								
**BQggg8494**	BQggg8494_30.1	=HM235041							BQggg8494_5.1	KU665652
	BQggg8494_30.2	=HM234986							BQggg8494_5.2	=HM235148
	BQggg8494_30.7	=HM234984							BQggg8494_5.4	=HM235154
	BQggg8494_30.11	=HM235064								
**BQggg8495**	BQggg8495_5.1	=HM235069							BQggg8495_5.1	=HM235148
	BQggg8495_5.4	KU665671								
	BQggg8495_5.5	=HM235037								
	BQggg8495_5.6	=KT824293								
	BQggg8495_5.12	=HM234986								
**BQggg8746**	BQggg8746_10.11	=HM234986							BQggg8746_5.6	=HM235167
**BQggg8750**	BQggg8750_5.9	=BQggg8495_5.4								
**BQggg8752**	BQggg8752_5.2	KU665672			BQggg8752_10.7	=KF568920				
	BQggg8752_10.7	=HM234986								
**BQggg8758**	BQggg8758_10.4	=KT824293							BQggg8758_5.6	=HM235148
	BQggg8758_10.9	=HM235066								
**BQggg8764**	BQggg8764_10.2	=HM234986								
	BQggg8764_10.3	=HM235041								
	BQggg8764_10.7	=KT824293								
	BQggg8764_10.10	=BQggg8752_5.2								
	BQggg8764_10.12	=HM235058								
**BQggg8768**	BQggg8768_10.1	=HM234986							BQggg8768_5.4	=HM235148
	BQggg8768_10.3	=HM235017								
	BQggg8768_10.9	=BQggg8752_5.2								
**BQggg8770**	BQggg8770_10.5	=HM234986								
**BQggg8771**	BQggg8771_10.1	=HM235069	BQggg8771_5.6	KT824324					BQggg8771_5.4	=HM235148
	BQggg8771_10.3	=HM235037								
	BQggg8771_10.10	=HM234986								
	BQggg8771_10.11	KT824287								
**BYggg3814**	BYggg3814_1.1	=HM234988								
**BYggg3821**	BYggg3821_5.1	=HM235041								
**BYggg5016**	BYggg5016_1.1	KU665673								
**BYggg5029**	BYggg5029_1.1	KU665674								
**CPggg6665**	CPggg6665_5.9	=HM235066								
**CPggg6673**	CPggg6673_5.7	=HM234986								

**CPggg9521**	CPggg9521_10.12	=HM235064										
**CPggg9524**	CPggg9524_10.12	=HM235064										
	CPggg9524_10.6	KU665675										
**CPggg9528**											CPggg9528_5.2	=HM235154
**CPggg9529**	CPggg9529_5.4	=HM234988										
	CPggg9529_10.9	KU665676										
**CPggg9536**	CPggg9536_30.9	=KT824289									CPggg9536_5.8	=HM235165
**CPggg9664**							CPggg9664_10.12	=GTggg119_10.10			CPggg9664_5.3	=HM235154
**CPggg9668**	CPggg9668_5.8	=KT824289										
**CPggg9669**	CPggg9669_10.6	=CPggg9529_10.9										
**CPggg9671**	CPggg9671_5.1	=KT824289										
**CPggg9678**	CPggg9678_5.12	=KT824289										
**CPggg9681**	CPggg9681_10.8	=HM235064										
**CPggg9682**	CPggg9682_10.4	=CPggg9529_10.9										
**CPggg9687**	CPggg9687_10.2	=HM235041										
**CPggg9850**	CPggg9850_10.4	=HM235064									CPggg9580_5.5	=HM235154
**DDggg27**							DDggg27_2.1	KU665748				
**DDggg3567**	DDggg3567_5.1	=HM235064			DDggg3567_5.8	=KF568921						
**DDggg3568**	DDggg3568_5.3	=HM235064										
**DDggg3603**	DDggg3603_5.1	=HM235064										
**DDggg3606**	DDggg3606_1.1	=HM235041										
**DDggg3608**	DDggg3608_1.1	=HM235064										
	DDggg3608_5.5	KU665677										
**DDggg4327**	DDggg4327_5.1	=HM234986										
**DDggg4331**	DDggg4331_5.1	=HM235064			DDggg4331_5.5	=KF568921						
**DDggg4334**	DDggg4334_1.1	=KT824293										
**DDggg4346**	DDggg4346_5.5	=HM234986										
**DSggg24**									DSggg24_5.1	=HM235140		
**DSggg60**							DSggg60_2.3	KU665750				
**GTggg118**					GTggg118_10.1	=KF568920	GTggg118_2.23	=BQggg61_2.7				
							GTggg118_10.3	KU665751				
**GTggg119**							GTggg119_10.10	KU665752			GTggg119_80.2	=HM235154
**GTggg509**							GTggg509_2.9	KU665753	GTggg509_5.11	KU665721	GTggg509_5.5	=HM235148
**GTggg8**			GTggg8_5.1	KT824335			GTggg8_2.1	=DDggg27_2.1				
							GTggg8_2.2	KU665754				
**GTggg10**					GTggg10_5.11	KU665710						
**GTggg833**											GTggg833_5.2	=HM235154
											GTggg833_5.8	=MBggg6756_5.4
**GTggg84**									GTggg84_5.2	=HM235120		
**GTggg85**											GTggg85_5.2	KU665653
											GTggg85_5.6	=HM235165
**LBggg1220**											LBggg1220_5.1	=HM235148
**LBggg1222**					LBggg1222_10.10	=KF568924	LBggg1222_10.10	KU665768			LBggg1222_5.3	=HM235148
											LBggg1222_5.6	KU665655
**LBggg177**							LBggg177_2.7	KU665769				
**LBggg314**							LBggg314_2.1	=BQggg60_2.1				
							LBggg314_2.2	KU665770				
							LBggg314_2.11	KU665771				

**LMggg1671**									LMggg1671_5.9	KU665723	LMggg1671_5.1	=HM235165
											LMggg1671_5.3	=HM235148
**LMggg1672**			LMggg1672_5.6	KT824351							LMggg1672_5.4	=HM235148
**MBggg5324**	MBggg5324_5.1	=MBggg6776_10.2									MBggg5324_5.1	=HM235148
	MBggg5324_5.3	=KT824293									MBggg5324_5.4	=HM235167
	MBggg5324_5.10	KU665685										
**MBggg6746**	MBggg6746_5.11	=HM235037										
	MBggg6746_10.12	=HM234986										
**MBggg6756**	MBggg6756_30.1	=HM235041									MBggg6756_5.4	KU665656
											MBggg6756_5.6	KU665657
**MBggg6776**	MBggg6776_10.2	KU665686									MBggg6776_5.2	=HM235164
**MMggg1511**	MMggg1511_5.12	=HM235064										
**MMggg1562**	MMggg1562_5.1	KU665687									MMggg1562_5.7	=HM235165
	MMggg1562_5.6	=HM235041										
	MMggg1562_5.10	=HM234986										
**MMggg1598**	MMggg1598_5.10	KU665688										
**MMggg1601**	MMggg1601_5.2	KU665689										
	MMggg1601_5.8	=HM234986										
	MMggg1601_5.9	=HM234987										
**MMggg1603**	MMggg1603_5.1	=HM235041										
**MMggg1604**	MMggg1604_5.5	=HM234986									MMggg1604_5.4	KU665658
	MMggg1604_5.11	=MMggg1562_5.1									MMggg1604_5.8	=HM235165
	MMggg1604_30.12	=HM235041										
**MMggg1607**	MMggg1607_30.5	=HM234986									MMggg1607_5.7	=HM235165
**MMggg1608**	MMggg1608_30.7	=HM235041									MMggg1608_5.3	=HM235165
**MMggg1609**	MMggg1609_5.11	=HM234986										
**MMggg1614**	MMggg1614_5.5	=HM234986										
	MMggg1614_5.10	=HM235066										
**MMggg1616**	MMggg1616_30.9	=HM235041									MMggg1616_5.2	=MMggg1604_5.4
**MMggg1622**	MMggg1622_5.6	=HM235041										
**MMggg1624**	MMggg1624_5.5	=HM235041									MMggg1624_5.4	=HM235165
**MSggg7183**	MSggg7183_30.2	=HM235064										
	MSggg7183_30.9	=HM235041										
**MSggg7193**	MSggg7193_5.7	=HM234986										
**MSggg7212**	MSggg7212_5.10	KU665690										
	MSggg7212_5.12	=HM234986										
**NDggg3203**							NDggg3203_2.2	KU665777				
							NDggg3203_2.4	KU665778				
							NDggg3203_2.5	KU665774				
							NDggg3203_2.7	KU665779				
							NDggg3203_10.14	KU665775				
							NDggg3203_10.35	KU665776				
**NGggg4475**	NGggg4475_1.1	=HM234986										

[a]Single template amplified loci of *Laverania* mitochondrial (*cytB*), nuclear (*eba165, eba175, p47, ldh*), and apicoplast (*clpM*) genes (the *clpM* gene, which encodes the Clp chaperone PfC10_API0060, has previously been called *clpC;* Liu et al. 2010a).
[b]New GenBank accession numbers are highlighted in yellow.

Supplementary References

Cummings MP, Neel MC, Shaw KL. 2008. A genealogical approach to quantifying lineage divergence. Evolution 62:2411-2422.

Liu W, et al. 2010. Origin of the human malaria parasite *Plasmodium falciparum* in gorillas. Nature 467:420-425.

Sundararaman SA, et al. 2016. Genomes of cryptic chimpanzee *Plasmodium* species reveal key evolutionary events leading to human malaria. Nat Commun., *in the press*.

Wanaguru M, Liu W, Hahn BH, Rayner JC, Wright GJ. 2013. RH5-Basigin interaction plays a major role in the host tropism of *Plasmodium falciparum*. Proc Natl Acad Sci U S A. 110:20735-20740.
